# Bacterial stress responses lower mRNA–protein level correlations

**DOI:** 10.1073/pnas.2613102123

**Published:** 2026-09-16

**Authors:** Sena G. Süer, Jérôme Arnoux, Yi Y. Lim, Ganeshwari Dhurve, Rabia Şen, Cemal Erdem, André Mateus, Kemal Avican

**Affiliations:** ^a^ Department of Molecular Biology, Umeå University, Umeå 90187, Sweden; ^b^ Integrated Science Lab (IceLab), Umeå University, Umeå 90187, Sweden; ^c^ Umeå Centre for Microbial Research (UCMR), Umeå University, Umeå 90187, Sweden; ^d^ Science for Life Laboratory (SciLifeLab), Umeå University, Umeå 90187, Sweden; ^e^ Laboratory for Molecular Infection Medicine Sweden (MIMS), Umeå University, Umeå 90187, Sweden; ^f^ Department of Medical Biosciences, Umeå University, Umeå 90187, Sweden; ^g^ Department of Chemistry, Umeå University, Umeå 90187, Sweden

**Keywords:** protein, mRNA, bacteria, stress response, data integration

## Abstract

Diverse bacterial pathogens have evolved complex regulatory mechanisms to adapt to various environmental stresses during infection. The uncertainty in mRNA–protein levels in response to environmental stressors complicates our understanding of bacterial physiology and their adaptation to stressful environments. To examine this issue, we have integrated transcriptomics and proteomics data on three human bacterial pathogens: *Salmonella enterica* Typhimurium, *Yersinia pseudotuberculosis*, and *Staphylococcus aureus* under 10 infection-relevant stress conditions. We observed positive correlations between mRNA and protein levels, which were decreased under different stress conditions. Essential genes exhibited higher expression levels with lower variation across the conditions and stronger mRNA–protein correlations compared to nonessential genes, highlighting their critical role in bacterial adaptability and survival. Moreover, we identified a substantial number of genes with stress-induced noncorrelating mRNA–protein levels, particularly under conditions triggering strong stress responses. Particularly this level was dramatically lowered for osmotic stress-specific genes affected by impaired translational activity under osmotic stress. Our findings highlight the prevalence of noncorrelating mRNA–protein levels and the potential role of posttranslational modifications in modulating protein levels in response to environmental stressors during infection. This study provides a comprehensive framework for integrating transcriptomics and proteomics data and identifies potential gene products that might significantly impact the ability of diverse bacterial pathogens to adapt to hostile infection environments.

## Significance

We employed an integrative transcriptomics and proteomics approach to investigate mRNA–protein correlations in three clinically relevant pathogens under 10 infection-relevant stress conditions. We identified genes whose mRNA–protein correlations are systematically altered under stressors, pronounced by a subset of dynamically regulated genes representing active stress responses. This study provides a deeper understanding of mRNA–protein level uncertainty, coupling it to stress responses known to trigger critical posttranscriptional and posttranslational regulations, filling a gap that is identified in the field. These findings help reveal unknown mechanisms of bacterial adaptation and pathogenesis. By providing a comprehensive, multispecies dataset, this study serves as a foundational resource for infection biology, driving future advancements in understanding complex regulatory networks and identifying potential antimicrobial targets.

Bacterial pathogens have evolved complex mechanisms to adapt to different environmental stresses in the host such as nutrient limitations, temperature or pH shifts, and antimicrobial challenges during infection. They respond to deleterious effects of such stressors via stress response mechanisms, involving complex gene regulatory networks, for survival. Therefore, comprehensive understanding of bacterial stress response mechanisms is critical for development of novel therapeutic approaches to resolve bacterial infections. One useful approach is through investigation of global transcriptomes of diverse bacterial pathogens under a variety of stress conditions that mimic host environments. This provides information on regulation across diverse stress conditions that could be used in attempts to reveal complex gene regulations and identify key stress response genes. We previously performed a large-scale transcriptomic study of 32 human bacterial pathogens, PATHOgenex RNA Atlas (1), aiming to understand bacterial stress responses under 11 in vivo mimicking stress conditions. The study revealed a dynamic regulation of gene expression under different stress conditions and suggested a set of genes that has potential to be candidate as antimicrobial targets. Co-PATHOgenex (2), the continuation of the PATHOgenex study, identifies stress-specific stimulons by generating gene coexpression modules. These stimulons represent sets of genes with unique expression patterns under specific stressors, providing insights into the stress adaptation to different stressors.

Yet the main functional units shaping the cellular response are proteins and there is, generally, a weak correlation between mRNA and protein levels (3–11). This highlights the complex nature of gene regulation and the diverse posttranscriptional factors affecting protein stability and abundance (12). It is known that transcription and translation could be uncoupled constitutively or under certain conditions, such as stress conditions, at a global or at a gene-specific level (13, 14). Therefore, the relationship between mRNA and protein levels can vary depending on the conditions, and this variability has been the subject of extensive research and ongoing debate (12, 14–16). Nevertheless, mRNA levels have been shown to be positively correlated with protein levels in many different organisms (8, 17–26). Linking protein expression to gene expression can help to identify key proteins in response to specific stimuli or conditions, and highlight mechanisms that drive bacterial adaptation, evasion of the immune system, and disease development (27–31). This is crucial for developing more effective diagnostic tools, treatment strategies, and vaccines for bacterial infections (32, 33). Therefore, integrated transcriptomic and proteomic studies have emerged as powerful tools for understanding the complex molecular mechanisms underlying bacterial pathogenesis (29). Despite the numerous studies integrating transcriptome and proteome data, efforts to identify genes where mRNA and protein levels do not correlate are relatively limited (17, 34). Focusing on such genes could offer valuable insight into the complex posttranscriptional and posttranslational regulatory mechanisms that escape detection by analyses addressing correlated gene-expression patterns, specifically under infection-relevant stress conditions.

In this study, we employed an integrative transcriptomic and proteomic analysis of *Salmonella enterica* Typhimurium, *Yersinia pseudotuberculosis*, and *Staphylococcus aureus* under 10 infection-relevant stress conditions to investigate mRNA–protein level correlations. We revealed that mRNA–protein correlations significantly varied under different stress conditions for the three species. Essential genes consistently showed higher mRNA and protein expression levels and stronger correlations, emphasizing their importance in bacterial survival. Stress conditions such as hypoxia and nutritional downshift exhibited the lowest correlations, suggesting that posttranscriptional and posttranslational regulation play a significant role under these conditions. We identified plentiful genes that showed noncorrelating mRNA–protein levels in the three species, as well as sets of genes that were species specific, particularly under conditions eliciting strong stress responses. Among all conditions tested, osmotic stress stood out as exhibiting the most pronounced mRNA–protein decoupling. Using the MOBILE (35) framework, we characterized the osmotic stress stimulon and linked the observed mRNA–protein decoupling to a specific impairment of translation initiation.

## Results

### Transcriptomic and Proteomic Analysis of Three Human Bacterial Pathogens Under 10 Infection-Relevant Stress Conditions.

**Fig. 1.**
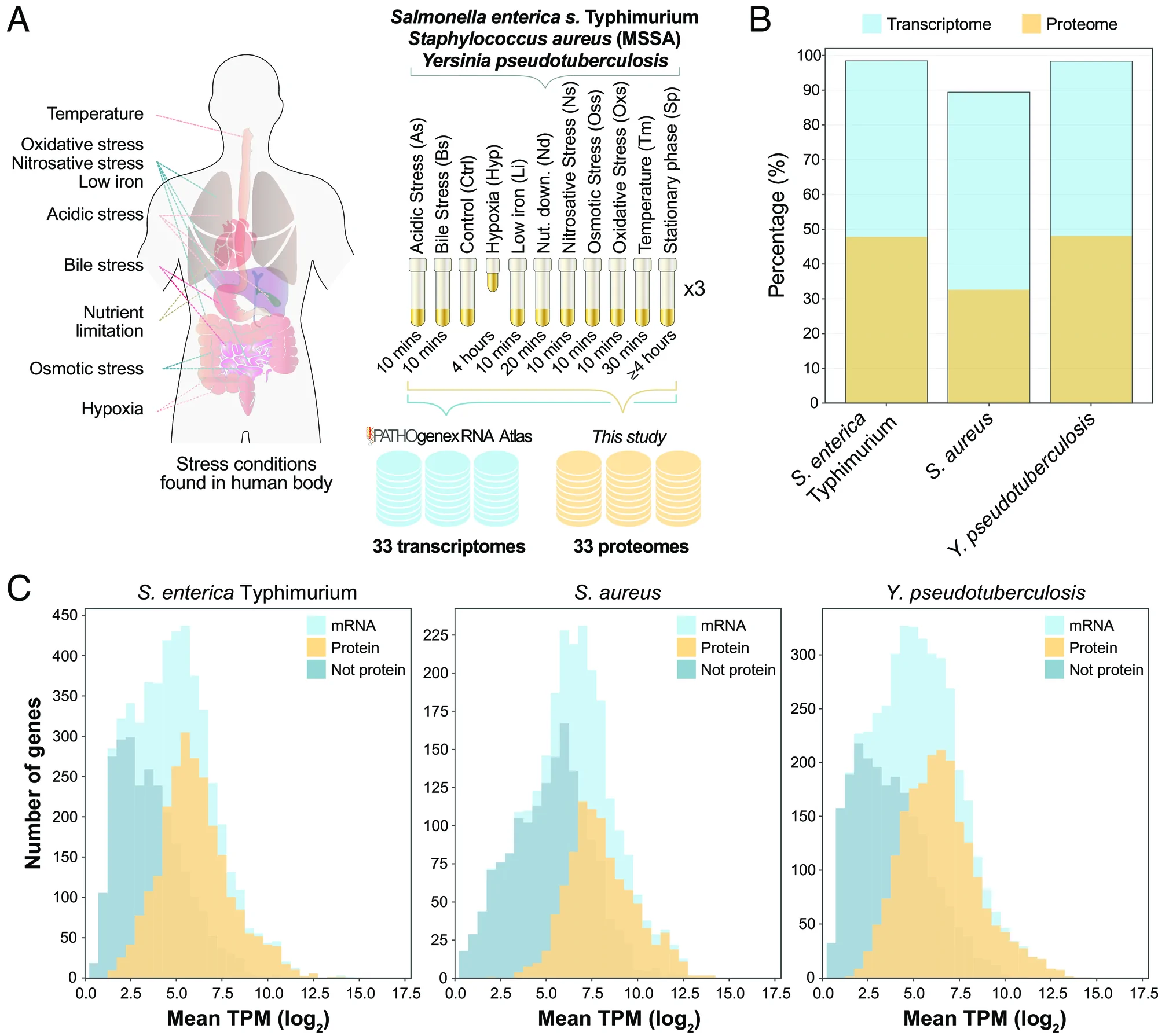
Transcriptomic and proteomic analysis of three human bacterial pathogens under 10 infection-relevant stress conditions. (*A*) Schematic presentation of data collection and data generation for gene expression on mRNA and protein levels under stress conditions. (*B*) Percentages of CDSs detected by transcriptomics and proteomics for the three species. (*C*) Distribution of transcript abundance across all conditions (light blue) in the three species; yellow indicates the fraction of proteins detected, while dark blue represents the transcripts for which no corresponding protein was detected.

We analyzed transcriptomic and proteomic datasets of three human bacterial pathogens including Gram-negative *S. enterica* Typhimurium SL1344, *Y. pseudotuberculosis* YPIII and Gram-positive *S. aureus* MSSA476. We used transcriptomes of the three pathogens under 10 infection-relevant stress conditions together with unexposed control condition from PATHOgenex RNA Atlas (1) and complemented it with mass-spectrometry proteomic profiling under the same stressors (Fig. 1*A*). The 10 conditions are mimicking stresses that bacteria encounter in the human host: acidic stress (the low pH of the stomach and phagosome), bile stress (the intestinal and biliary tract), osmotic stress (the hyperosmolarity of blood and the gut lumen), temperature (the thermal shift upon host entry and fever), low iron and nutritional downshift (host-imposed iron and nutrient limitation), oxidative and nitrosative stress (the reactive oxygen and nitrogen species generated by immune cells), hypoxia (oxygen-poor niches such as the gut lumen and inflamed or abscessed tissue), and stationary phase (growth arrest under nutrient exhaustion). Apart from hypoxia and stationary phase stress corresponding to 4-h exposures, the other eight stresses are acute shocks of 10 to 30 min.

As mass spectrometry-based proteomics often yields a lower coverage than RNA-seq, we compared transcripts and proteins detected across all conditions in three pathogens, focusing on protein coding sequences (CDSs) in the analysis. While almost all CDSs were detected by RNA-seq for the three pathogens (98% for *S. enterica* and *Y. pseudotuberculosis*, and 90% for *S. aureus*), proteomics could detect 48% of the CDSs for *S. enterica* Typhimurium and *Y. pseudotuberculosis*, and 32% for *S. aureus* (Fig. 1*B*). Detected proteins covered nearly the full spectrum of mRNA expressions, with a bias toward highly expressed mRNAs across the conditions for the three pathogens (Fig. 1*C*).

### mRNA and Protein Level Correlation Under Stress.

**Fig. 2.**
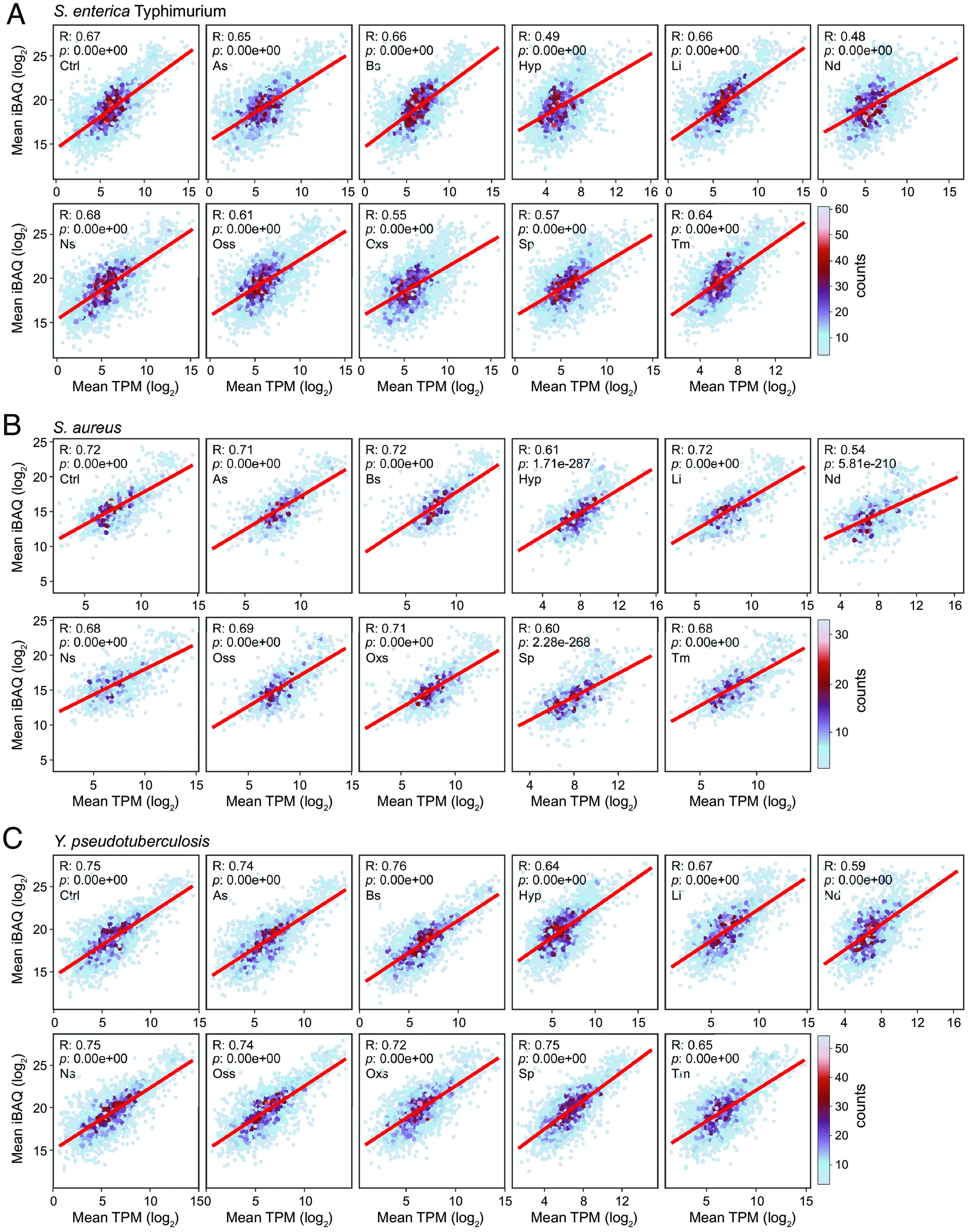
mRNA–protein levels show differing degrees of statistically significant positive correlation under different stress conditions and control conditions in (*A*) *S. enterica* Typhimurium, (*B*) *S. aureus*, and (*C*) *Y. pseudotuberculosis*. mRNA and protein expression levels were taken as log_2_ transformed TPM and iBAQ values, respectively. The expression values of genes, that are detected in all conditions, as mRNA and protein were used in this analysis. Pearson correlation coefficient (R) shows positive correlation under all conditions in the three species. *P*-values were calculated using Student’s *t* test.

The correlation between mRNA and protein levels has been previously explored in bacteria under individual growth conditions (18, 34, 36, 37). Our data allow us to assess whether mRNA levels can serve as an accurate indicator of protein levels in three human bacterial pathogens. To evaluate how different stressors affect the mRNA–protein level correlation, we calculated the intensity-based absolute quantification (iBAQ) (15) (Dataset S1) values for protein levels and compared to mRNA levels in transcript per million (TPM) (38) for every stress condition and the unexposed control condition (1). We have previously shown that the three biological replicates of the RNA-seq samples clustered together (1). For the proteome dataset generated in this study, we conducted pairwise comparisons of the three replicates of each condition and computed Pearson correlation coefficient values (R-value). The R-value for pairwise comparison within the replicates of the same condition varied from 0.89 to 0.96 for the three pathogens (*SI Appendix*, Figs. S1–S3) showing strong similarities between the replicates. We then measured the correlation between mRNA and protein levels by calculating Pearson correlation coefficient values of the log_2_ transformed mean TPM and log_2_ transformed mean iBAQ of genes for each condition and pathogens (Fig. 2 *A–C*). For these measurements, we limited the analysis to the genes that are detected as mRNA and protein across all tested conditions. We observed that correlations between mRNA and protein levels under 11 conditions ranged from 0.48 to 0.68 for *S. enterica* Typhimurium, 0.54 to 0.72 for *S. aureus* and 0.59 to 0.76 for *Y. pseudotuberculosis* (Fig. 2 *A–C*). These correlation coefficients are comparable to those reported in previous studies across diverse biological systems (19, 20, 34, 37, 39). The variation in mRNA–protein correlation across stress conditions suggests that distinct molecular mechanisms, activated by each stressor, might modulate translational or posttranslational regulations leading to differences.

To further characterize the nature of stress-induced expression changes, we compared the correlation of each stress condition with its respective unexposed control, separately for transcriptomes and proteomes (*SI Appendix*, Fig. S4). Across all three species, proteomes consistently showed higher correlation with the control condition than transcriptomes. Notably, hypoxia and stationary phase show a lower mRNA correlation with control than other stress conditions (0.76 to 0.80 and 0.66 to 0.88, respectively), but also the lowest protein correlation (0.96 to 0.97 and 0.94 to 0.98, respectively). These results indicate that stress response impacts mRNA more than protein. Of note, even under short exposure conditions such as nutritional downshift, where protein levels overall remained stable (0.98 to 0.99), a subset of proteins remain uncorrelated with control. This suggests that abundance of a part of the protein content is dynamically and rapidly changing. Additionally, while many stress conditions show a similar expression profile with control (on the diagonal dashed line), certain conditions in specific species show a different profile with control. For the three species, stationary phase or hypoxia presents the biggest difference, but temperature for *S. enterica*, nutritional downshift for *S. aureus* and oxidative stress as well as low iron for *Y. pseudotuberculosis* present also a higher visual difference compared to other stresses. This suggests that some conditions, regardless of exposure time, present an overall change in protein abundance in comparison to control.

### Essential Genes Have Higher mRNA–Protein Level Correlations in the Three Species.

**Fig. 3.**
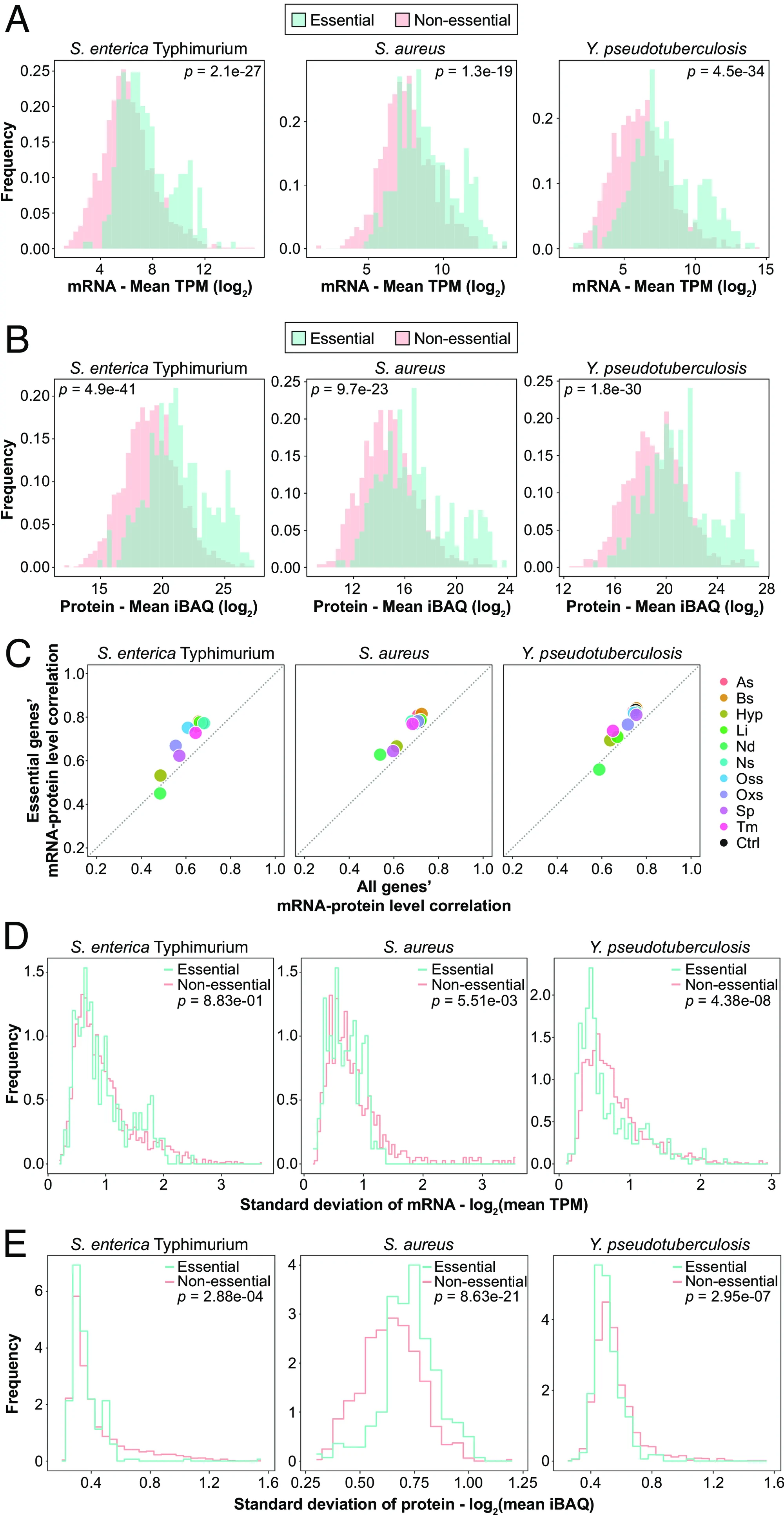
Essential genes of the three pathogens have higher mRNA and protein expressions with higher correlation and lower variance across the 11 conditions in comparison to all genes. (*A*) Mean mRNA expression frequency of essential and nonessential genes in the 11 conditions. (*B*) Mean protein expression frequency of essential and nonessential genes in the 11 conditions. (*C*) Essential genes and all genes mRNA–protein level correlation under 11 conditions for the three species. (*D*) Frequency of SD of mRNA expression under 11 conditions for the three species. (*E*) Frequency of SD of protein expression under 11 conditions for the three species. mRNA–protein level correlations were measured with Pearson correlation coefficient (R). *P*-values were calculated using a Wilcoxon rank sum test.

The positive correlation between mRNA–protein levels for all detected genes prompted us to investigate subsets of genes with different correlations levels. Essential genes have been shown to have stronger correlations in comparison to nonessentials for *Pseudomonas aeruginosa* during different growth phases (17). Therefore, we sought to investigate mRNA–protein level correlation profiles of essential genes in the three species. Of the 354, 341, and 445 previously identified essential genes for *S. enterica* Typhimurium (40), *S. aureus* (41), and *Y. pseudotuberculosis* (42), respectively, we detected 300 (85%), 280 (82%), and 335 (75%) essential genes in our transcriptome and proteome datasets. We found that mean expression levels of mRNAs and proteins were significantly higher for essential genes in comparison to nonessentials for the three species (Fig. 3 *A* and *B*). This was true for the expression levels in every condition (*SI Appendix*, Figs. S5 and S6). Moreover, we observed that essential genes have higher mRNA–protein level correlation than all detected genes for all three species (Fig. 3*C*), except for *S. enterica* Typhimurium and *Y. pseudotuberculosis* under nutritional downshift stress condition. As previously observed in other species (43, 44), essential genes showed significantly lower variance on both mRNA and protein expression across the 11 conditions for the three species (Fig. 3 *D* and *E*). These findings show that essential genes of *S. enterica* Typhimurium, *S. aureus*, and *Y. pseudotuberculosis* have high and stable mRNA and protein expression under a wide range of stress conditions with robust mRNA–protein level correlations.

Comparing stress and control conditions across all three species, essential genes consistently showed higher correlation for mRNA and protein than all genes combined (*SI Appendix*, Fig. S7). As expected from our previous results (Fig. 3 *D* and *E*) and their constitutive role in cellular physiology, essential genes were less transcriptionally and translationally regulated under stress than nonessential genes, maintaining stable expression at both the RNA and protein level. This stability prompted us to focus on the genes that are specifically and dynamically regulated under stress conditions.

### Stress Responses Differentially Affect mRNA–Protein Correlation.

To assess whether the degree of stress responses through differentially regulated mRNAs and proteins has an impact on mRNA–protein level correlations, we used differentially expressed genes (DEGs) and differentially expressed proteins (DEPs) for each condition in the three species by using unexposed samples as control. Before identifying DEPs, we evaluated eight normalization methods from NormalyzerDE (45), on the iBAQ protein abundance data and found that variance stabilizing normalization (VSN) was the most effective in clustering the condition together (*SI Appendix*, Fig. S8). Then we used limma (46) differential expression package for the identification of DEPs with VSN normalized data. For DEGs, we used previously reported differential expression analysis of PATHOgenex RNA Atlas (1). We employed a *P*-value < 0.05 and a log_2_-Fold Change > |0.5| as thresholds to identify DEGs and DEPs for each condition.

**Fig. 4.**
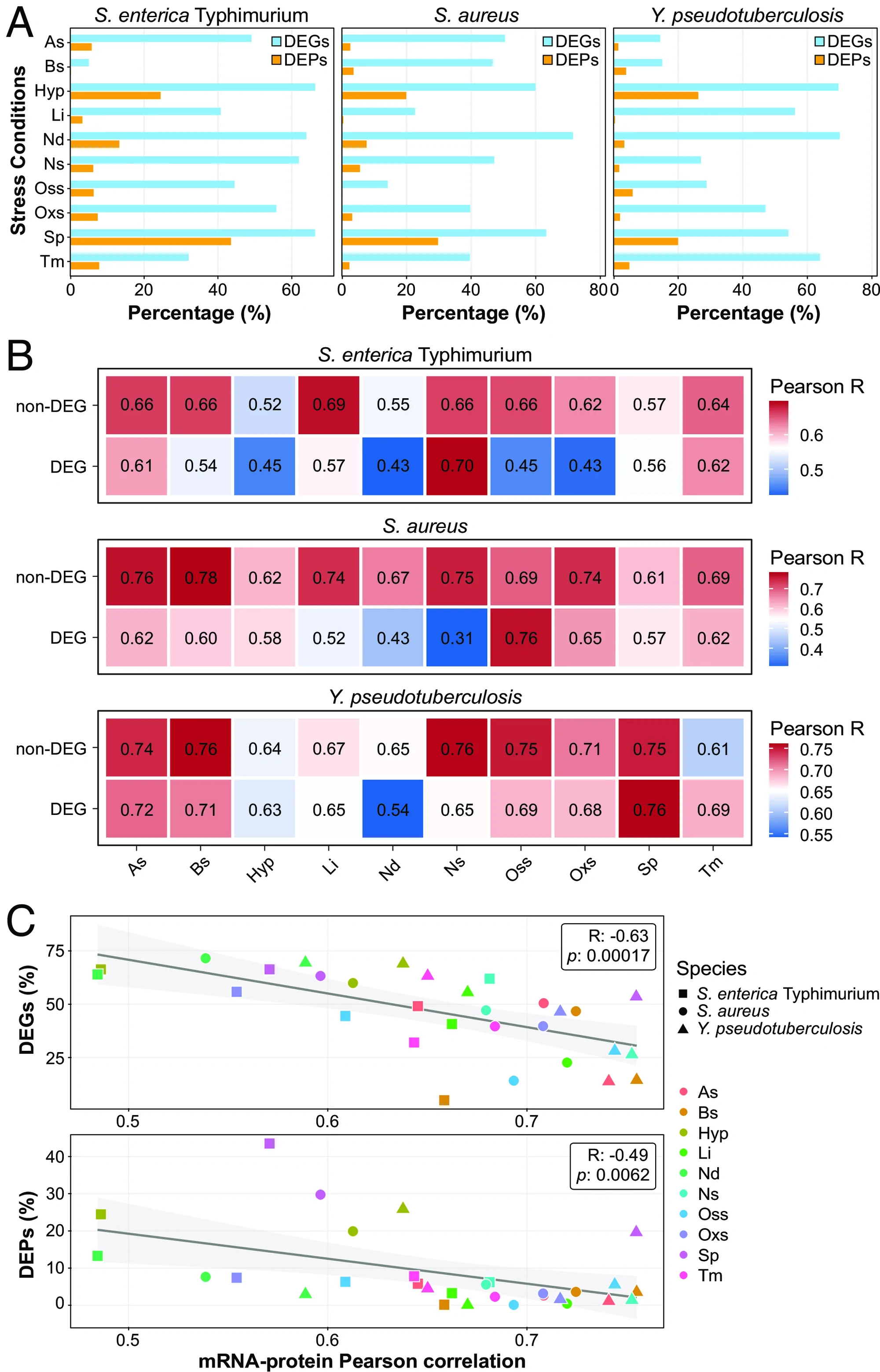
mRNA–protein level correlation lowers with increasing degree of stress response. (*A*) Percentages of DEGs and DEPs under the 10 stress conditions in comparison to unexposed control conditions for the three species. (*B*) Heatmap DE genes and all genes mRNA–protein level correlation under 10 conditions for the three species. (*C*) Scatter plots showing negative correlation between percentages of DEGs (*Top*) and DEPs (*Bottom*) and mRNA–protein level correlation. Correlations were measured with Pearson correlation (R) for (*B* and *C*). *P*-values were calculated using Student’s *t* test.

Notably, the number of DEGs was significantly higher compared to DEPs in all conditions for the three species (Fig. 4*A*). This can be linked to the stronger regulation of mRNA levels than of protein levels as previously observed in humans (16, 47) or the buffering effect of protein level (*SI Appendix*, Fig. S4). Moreover, percentages of DEGs and DEPs were highest under hypoxia, nutritional downshift, and stationary phase conditions for the three pathogens (Fig. 4*A*).

We then examined the mRNA–protein correlation of DEGs (Fig. 4*B* and *SI Appendix*, Fig. S9). Across all species and stress conditions, DEGs consistently showed lower mRNA–protein correlation than all genes, except for stationary phase and high temperature in *Y. pseudotuberculosis* (0.75 vs. 0.76 and 0.65 vs. 0.69, respectively). This supports our hypothesis that the reduction in mRNA–protein correlation is not a global phenomenon but rather reflects dynamic and active regulation in a specific subset of genes. A similar trend was observed for DEPs (*SI Appendix*, Fig. S10); however, the low number of DEPs across conditions limits our ability to draw equivalent conclusions.

Next, we tested whether the differential expressions have an impact on mRNA–protein level correlations. To do this, we measured the association between the percentages of DEGs and DEPs and the mRNA–protein level correlations with Pearson correlation coefficient. Our results revealed a strong negative correlation between both differential expressions and mRNA–protein level correlation (Fig. 4*C*), meaning that both DEGs and DEPs contribute to the discordance in correlation between mRNA and protein. This suggests that other factors, such as posttranscriptional and posttranslational regulations triggered by different stressors, might be influencing protein levels alongside the mRNA expression.

### Identification of Genes Whose mRNA–Protein Levels Are Discordantly Regulated Under Stress Conditions.

**Fig. 5.**
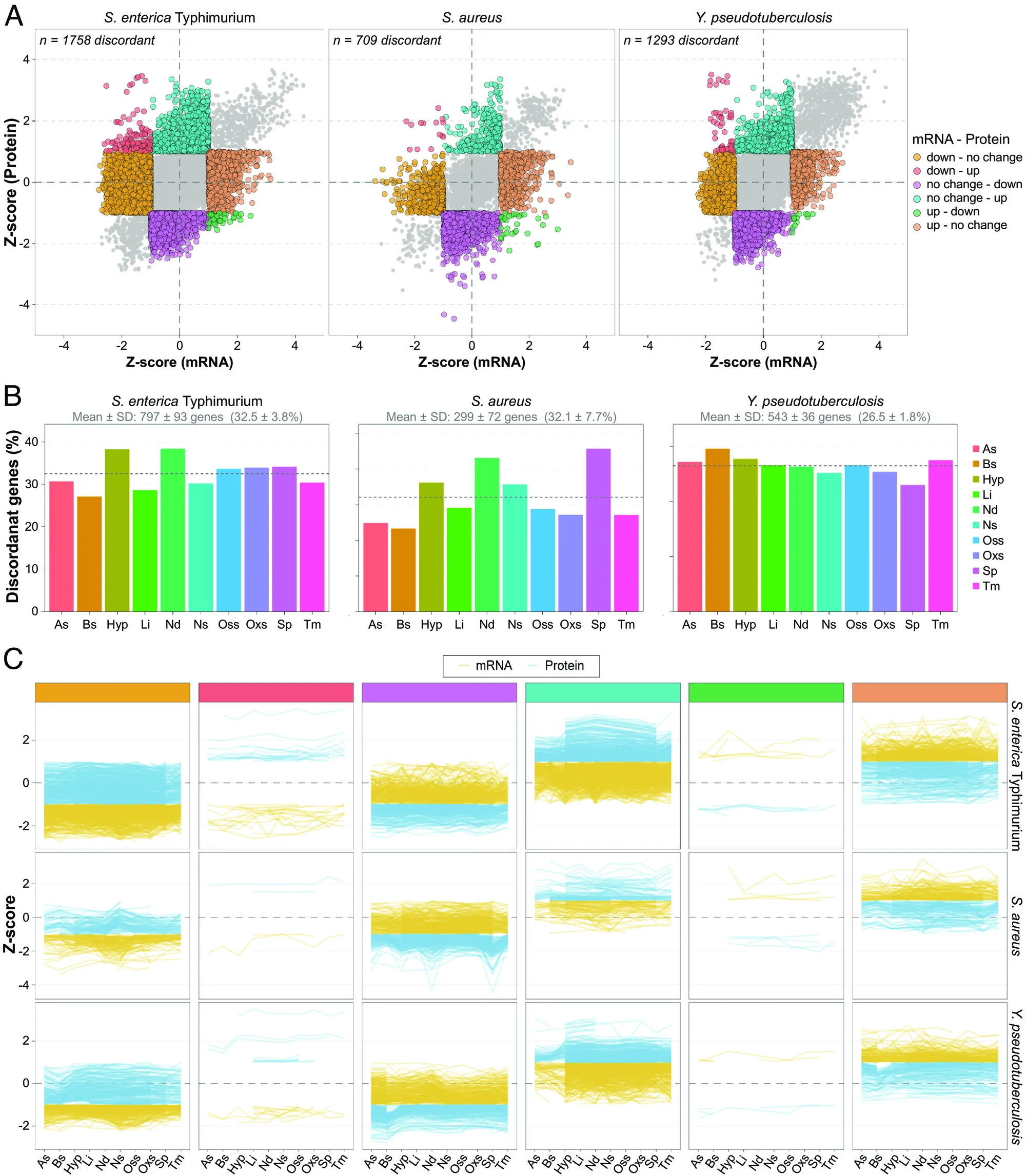
Stress response induces different patterns of discordant mRNA and protein level regulation. (*A*) Scatter plots with Z-scores of mRNA and protein levels for each of the 10 stress conditions in comparison to control for the three species. The 6 gene clusters whose mRNA and protein level have different discordant regulation patterns are shown in different colors. (*B*) Percentage of discordantly regulated genes per stress treatment. Numbers above bars indicate the exact gene count. The dashed line marks the mean percentage across all stress treatments for that species; the mean ± SD (in both gene counts and percentages) is reported in the subtitle. Bars are colored by treatment. (*C*) Z-score patterns of mRNA and protein levels across 10 stress conditions for the six discordantly regulated gene clusters in each species. The number of discordantly regulated genes per cluster is indicated with *n.*

The decreased correlation between mRNA–protein levels with increased number of DEGs and DEPs under specific stress conditions led us to investigate the genes whose mRNA–protein levels are discordantly regulated when the bacteria are exposed to stress conditions. We clustered genes into six groups (*SI Appendix*, Table S1) corresponding to distinct combinations of differential expression patterns at the mRNA and protein levels in at least one stress condition (Fig. 5 *A* and *C* and Dataset S2). We additionally quantified the number of discordantly regulated genes within each individual condition (Fig. 5*B*). On a per-condition basis, discordant genes represented a substantial and relatively uniform fraction of the detected genes in all three species (mean ± SD across conditions: 32.5 ± 3.8% [797 ± 93 genes] for *S. enterica* Typhimurium, 32.1 ± 7.7% [299 ± 72 genes] for *S. aureus*, and 26.5 ± 1.8% [543 ± 36 genes] for *Y. pseudotuberculosis*), with no single condition accounting for a disproportionate share. For each species, the sum of discordant genes across all conditions exceeded the number of unique discordant genes, indicating that many genes were discordant under more than one condition (*SI Appendix*, Fig. S11). We observed that 50% of the discordant genes are shared in at least four conditions for the three species. This overlap is expected, reflecting both a shared general stress response and the partial similarity between related stressors (e.g., hypoxia, nutritional downshift, and stationary phase). In agreement with lowest correlation between mRNA–protein levels observed under hypoxia, nutritional downshift, and stationary phase conditions (Fig. 2 *A*–*C*), discordantly regulated genes were mostly found under those conditions for the three species (*SI Appendix*, Fig. S12). Noteworthy, Clusters 1 and 6, where mRNAs were regulated but not proteins, had the highest number of discordantly regulated genes. This is in accordance with higher number of differentially regulated mRNAs (DEGs) compared to differentially regulated proteins (DEPs) in all conditions for the three species (Fig. 4*A*).

### Discordantly Regulated Genes Are Enriched in Translation.

To investigate whether the genes exhibiting discordant mRNA and protein levels under stress conditions are enriched in certain features, we performed Gene Ontology (GO) (48) enrichment analysis. Interestingly, we found that biological processes such as translation, RNA processing, RNA modification, phosphorylation, and cell division, among other biological processes were commonly enriched for the three species, particularly when mRNA levels were changed but there was no change at the protein level (*SI Appendix*, Fig. S13 and Dataset S3). This suggests that genes encoding subunits of translation machinery together with other components of translation such as tRNA and rRNA were discordantly regulated under stress conditions for the three pathogens. In detail, proteins in Cluster 1 have stable levels despite mRNA being downregulated, likely due to increased protein stability. Supporting this, we observed a cross-species enrichment of proteins involved in protein folding and posttranslational processing pathways. In contrast, proteins in Cluster 6 have stable levels despite mRNA being upregulated, suggesting reduced translation or increased proteolysis, which is enriched for *S. enterica* Typhimurium. Furthermore, we observed an enrichment in protein transport and secretion pathway for all species within this cluster. This indicates that the observed protein stability is not only due to translation and degradation activity but also to a rapid export from the cell. Moreover, genes encoding proteins involved in protein phosphorylation were commonly enriched for the three species in Cluster 4 and 5. In Cluster 4, protein levels were upregulated despite stable mRNA abundance, whereas Cluster 5 exhibited mRNA upregulation and protein downregulation (*SI Appendix*, Fig. S13). Notably, Cluster 4 was also commonly enriched for lipid metabolism across the three species. This indicates presence of posttranscriptional regulations such as regulation via small regulatory RNAs involved in carbon metabolism (49, 50) or posttranslational modifications which have high prevalence in bacterial metabolic enzymes (51).

### mRNA–Protein Level Stress-Specific Stimulon Genes Under Stress.

**Fig. 6.**
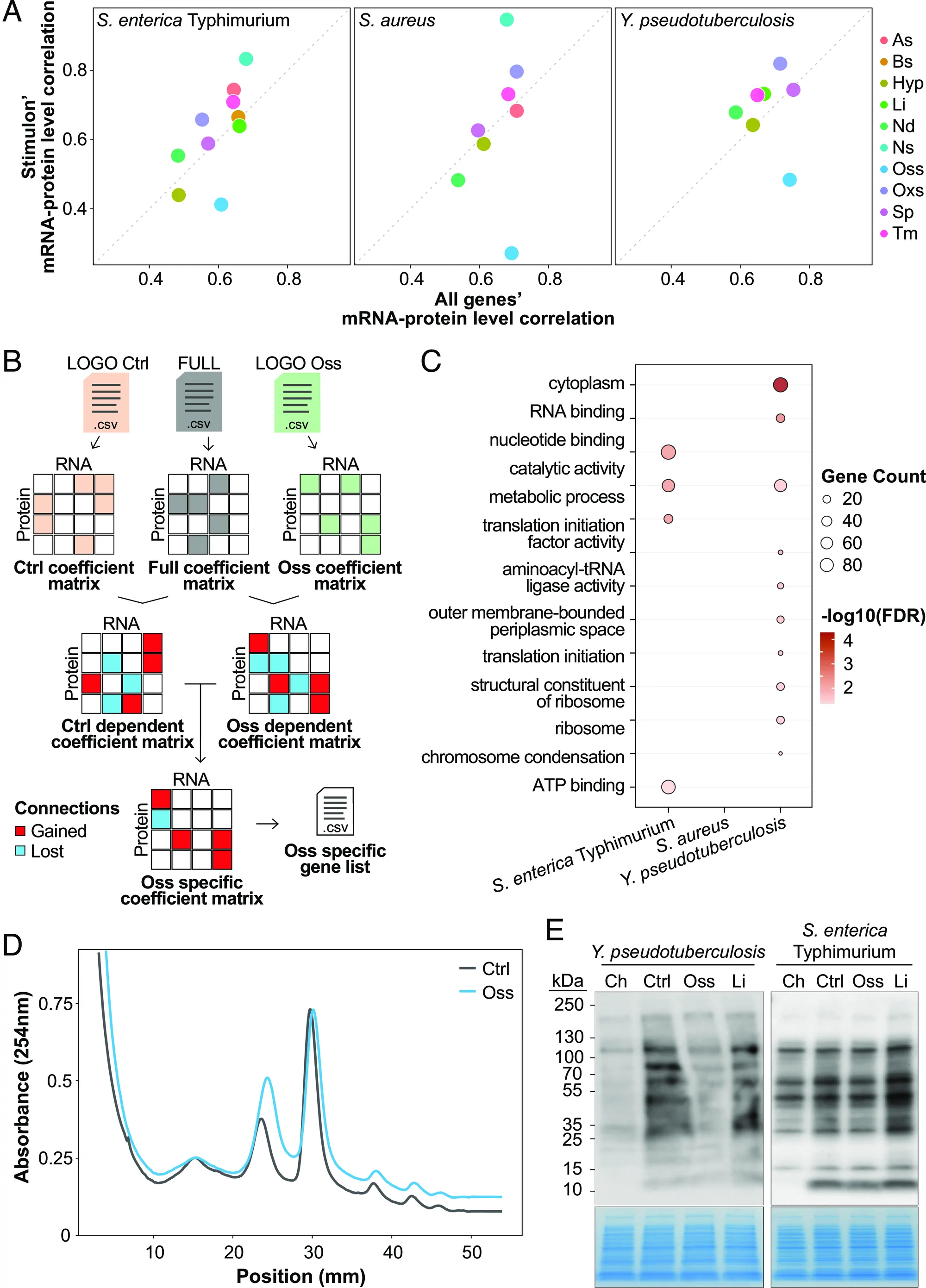
Impaired translation efficiency lowers mRNA–protein levels under osmotic stress. (*A*) Stress-specific stimulon genes and all genes mRNA–protein level correlation under 10 conditions for the three species. (*B*) MOBILE pipeline, (*C*) ORA analyses of osmotic stress-specific connections across three species, representing enrichment across biological process, molecular function, and cellular component. (*D*) Polysome profiles of wt *Y. pseudotuberculosis* cells under control (Ctrl) and osmotic stress (0.5 M NaCl) conditions. (*E*) Western blot membrane (*Upper* panels) and Coomassie blue–stained gel (*Lower* panels) of total proteins extracted from wt *Y. pseudotuberculosis* and *S. enterica* cells under negative control (Ch), positive control (Ctrl), osmotic stress (Oss), and low iron (Li). Active translation of nascent proteins was detected using a monoclonal anti-puromycin antibody.

We previously identified stress-specific stimulon genes as sets of genes that are uniquely coregulated in response to distinct stress conditions (2), representing a layer of regulation distinct from classical differential expression. Unlike DEGs, which can be differentially expressed across multiple conditions with similar patterns, stimulon genes are defined by their condition-specific expression signature (*SI Appendix*, Fig. S14). To assess whether these stimulon genes exhibit distinct mRNA–protein level correlations compared to other genes, we analyzed mRNA and protein differential expression data across all genes and stress-specific stimulons. No significant differences in mRNA–protein level correlations were observed between stimulon genes and other genes across most conditions except in the case of the osmotic stress-specific stimulon (Fig. 6*A* and *SI Appendix*, Fig. S15). Notably, genes within this stimulon showed a markedly lower mRNA–protein level correlation across all three pathogens studied.

To investigate this further, we used MOBILE framework (35) to identify osmotic stress-specific associations between mRNA and protein levels. Through repeated Lasso regression between paired omics layers, MOBILE enables the identification of condition-specific gene associations without requiring prior knowledge. The transcriptomic and proteomic datasets with all conditions were used as an input for MOBILE, yielding a list of statistically robust associations between proteins and mRNAs. Next, the osmotic stress condition data from both datasets were excluded from the input, and MOBILE is rerun to obtain condition-dependent associations list by comparing the all-data run to the latter. Similarly, the control condition data are held out, and MOBILE rerun yielded another list of associations to identify CTRL-data-dependent associations (Fig. 6*B*). We identified osmotic stress-specific associations by over representation analysis (ORA), comparing the osmotic stress-dependent and control-dependent association lists. Using osmotic stress-specific associations gene list, we ran a GO enrichment analysis. Interestingly, translation-related processes and components of the translational machinery were enriched only for *Y. pseudotuberculosis* (Fig. 6*C*). This suggests a functional link between osmotic stress-specific mRNA–protein decoupling and translational regulation. In contrast, *S. aureus* showed no significant enrichment in osmotic-specific pathways. This might reflect the inherent physiological resilience of Gram-positive bacteria and metabolic adaptation to osmotic stress (52).

Based on this finding, we hypothesized that the reduced mRNA–protein correlation observed for osmotic stress-specific stimulon genes might result from impaired translation under osmotic stress. To test this, we performed polysome profiling on *Y. pseudotuberculosis* exposed to osmotic stress for 10 min, using unexposed bacteria collected at matched time points as controls. The polysome profiles showed no notable differences between osmotic stress and control condition (Fig. 6*D*), indicating that global translation remains active under osmotic stress. However, global translational activity may be insufficient to meet the heightened translational demand triggered by the strong transcriptional induction of osmotic stress-specific stimulon genes.

To further characterize the translational response, translation efficiency was assessed using a puromycin incorporation assay across osmotic stress, low iron, and unexposed control conditions. Chloramphenicol-treated bacteria served as a translationally inactive control. Since puromycin causes a premature translation termination, its incorporation serves as a direct proxy for active translation. The results showed that translational activity was significantly reduced under osmotic stress compared to both unexposed control and low iron conditions, indicating that osmotic stress-specifically impairs translation activity (Fig. 6*E* and *SI Appendix*, Fig. S16). Therefore, the reduced mRNA–protein correlation for osmotic stress-specific stimulons is not surprising given the overall reduction in translational activity. These stimulon genes exhibit significant changes in mRNA abundance during stress. However, because the translational machinery is impaired, it cannot efficiently process or respond to these altered transcript levels. As a result, the correlation between mRNA and protein decreases. Notably, this decoupling is specific to osmotic stress and was not observed under low iron conditions. Similarly, *S. enterica* also had lowered translational activity under osmotic stress (not in low iron), which is surprising regarding MOBILE analysis which is not showing translational activity. Nevertheless, MOBILE identifies condition-specific mRNA–protein associations, indicating that the reduction in translation activity may not be exclusive to osmotic stress.

## Discussion

We conducted a comprehensive analysis by integrating transcriptomic and proteomic datasets of *S. enterica* Typhimurium, *Y. pseudotuberculosis*, and *S. aureus* under 10 infection-relevant stress conditions, as well as a control condition during exponential growth. The inclusion of diverse pathogens, which are distantly related yet possess conserved genes, enabled us to identify both common and species-specific mRNA–protein relationships across a range of conditions mimicking host environments. Consistent with prior findings in other organisms, our analyses indicate a positive correlation between mRNA and protein levels across the three bacterial pathogens. However, this correlation is significantly reduced under stress conditions, particularly those that trigger a strong stress response.

This reduction is not surprising, as stress response proteins might be involved in posttranscriptional or posttranslational regulations that can decouple mRNA and protein levels. Such regulations can occur through the stabilization or destabilization of mRNA and proteins as a response to the stress. RNA-binding proteins such as CsrA and ProQ can bind to target mRNAs under specific conditions, either stabilizing or destabilizing them, or preventing their translation (53–58). Furthermore, the discoordination of transcription and translation elongation rates under stress conditions may contribute to the decrease in mRNA–protein level correlation (59). For example, in *Escherichia coli* exposed to oxidative stress, there is a significant induction of mRNA expression of the OxyR regulon, which is crucial for survival under oxidative stress, while the translation elongation rate significantly slows down (60, 61). In contrast, under nutrient-limited conditions, there has been tight coordination between transcription and translation (62, 63). This suggests that the reduced mRNA–protein level correlation in our nutritional downshift condition might not be solely due to the discoordination of transcription and translation but rather other regulatory mechanisms. Conformational changes in riboswitches upon a change in the environment can also lead to reprogrammed mRNA translation and degradation, as is the case for the *lysC* riboswitch in the presence and absence of lysine (64).

Even though mostly studied in eukaryotes, posttranscriptional nucleotide modifications triggered by stress conditions can also have an impact on mRNA–protein level as it is shown for increased level of m^6^A methylation under hypoxia in human cancer cells (65). On the other hand, m^6^A methylation was shown to be lowered under increased temperature for *P. aeruginosa* (66). Similarly posttranslational modifications such as methylation, phosphorylation, acetylation, and sumoylation can directly impact protein stability or indirectly through triggered signaling cascade in both eukaryotes and prokaryotes (67, 68). Even though protein stability was not specifically investigated, it was shown that cellular protein phosphorylation was increased during *Streptomyces* differentiation to form spores, which is induced by stress conditions (69).

The mRNA–protein discrepancies observed are largely explained by changes in RNA expression, consistent with the kinetic buffering of the cellular protein pool (70). Our data aligned with this quantitative principle: protein levels remained more tightly correlated with the unexposed control than mRNA levels did, and acute stresses preserved this correlation better than longer exposures. Notably, a subset of proteins changes rapidly despite this general buffering, pointing to active regulation through accelerated turnover or posttranslational modification and representing the earliest proteomic signature of stress adaptation. Beyond this, DEGs emerge as the primary contributors to the decrease in mRNA–protein correlation, undergoing rapid transcriptional reprogramming while their protein levels remain largely unchanged over acute stress, thereby creating a transient but pronounced divergence. To assess whether the mRNA–protein discrepancies observed for DEGs are a common feature across species, we identified conserved DEGs across the three pathogens and compared their expression patterns. These conserved genes exhibited similar noncorrelating expression patterns under the same stress conditions, suggesting that posttranscriptional and posttranslational modifications affecting these genes may be conserved across species. This highlights the evolutionary significance of these regulatory mechanisms in bacterial adaptation to stress.

Among all conditions investigated for stress stimulons, osmotic stress exhibited the most pronounced mRNA–protein decoupling. This impairment may stem from structural remodeling of the cell envelope. Osmotic stress induces rapid cell depolymerization to manage turgor pressure (71, 72), which can physically disrupt membrane integrity and the efficiency of transmembrane transporters. In case of deficient methionine uptake, translation initiation would be affected due to the need of formylmethionine at the start of bacterial protein synthesis (21, 22). Consistent with this, translational activity was significantly reduced in *Y. pseudotuberculosis* under osmotic stress. The extension of the puromycin assay to *S. enterica* also revealed a reduction in translational activity under osmotic stress. However, unlike *Y. pseudotuberculosis*, this translational reduction was not identified as osmotic stress-specific by MOBILE, which might be explained by a common strategy as a response to different stress conditions, meaning that the translational response to osmotic stress is not exclusive to this condition but rather part of a broader stress regulon.

Our study underscores the importance of integrating transcriptomic and proteomic data to gain a holistic understanding of bacterial responses to infection-relevant stressors. The observed discrepancies between mRNA and protein levels emphasize the need for caution when inferring protein abundance solely from transcriptomic data. The precise posttranscriptional and posttranslational mechanisms contributing to the observed mRNA–protein discrepancies remain to be elucidated, as the currently available datasets do not allow their direct identification, representing an important avenue for future investigation.

In conclusion, our findings provide a comprehensive overview of mRNA–protein relationships under stress conditions in three bacterial pathogens, laying the groundwork for further investigations into the regulatory mechanisms mediating bacterial adaptation and survival in hostile host environments. The multispecies, multicondition nature of this dataset furthermore represents a valuable resource for training predictive models of protein abundance from mRNA levels, even across organisms by leveraging orthologous genes (26), particularly under stress conditions where the mRNA–protein relationship deviates substantially from baseline.

## Materials and Methods

### Bacterial Strains and Growth Conditions.

*S. enterica* subsp. *enterica* serovar Typhimurium SL1344, *S. aureus* subsp. *aureus* MSSA476, and *Y. pseudotuberculosis* serotype O:3 (strain YPIII) species were selected from PATHOgenex database (1) to analyze transcriptomic and proteomic data.

*S. enterica* SL1344 and *S. aureus* MSSA476 strains used in this study did not carry antibiotic resistance markers and were grown in Luria–Bertani (LB) medium (BD Difco™ LB Agar, Lennox) at 37 °C with shaking. The kanamycin-resistant wt *Y. pseudotuberculosis* (YPIII/pIBX) strain was used in this study. Unless otherwise stated, the wt *Y. pseudotuberculosis* strain was grown in LB medium supplemented with 50 µg/mL kanamycin at 26 °C with shaking. For puromycin experiments wt *S. enterica* was grown in LB medium supplemented with 50 µg/mL streptomycin at 37 °C with shaking.

### Stress Exposure and Sample Collection.

#### Polysome profiling.

The wt *Y. pseudotuberculosis* strain was grown overnight in LB, subcultured to an initial optical density (OD_600_) of 0.05 in 200 mL of LB. When the culture reached an OD_600_ of 0.3, the culture was divided into three 50 mL aliquots, each subjected to one of the following conditions for 10 min: 1) unexposed control, 2) osmotic stress (0.5 M NaCl), or 3) low iron stress (250 µM 2,2′-Bipyridyl; Sigma-Aldrich). Cells were harvested by filtration, immediately flash-frozen in liquid nitrogen, and stored at −80 °C until lysis.

Frozen cells were lysed by cryogenic grinding in liquid nitrogen using aluminum oxide. Lysis buffer was added during grinding, and the lysate was allowed to thaw gradually throughout the process. The mixture was transferred into a precooled 1.5 mL reaction tube and immediately placed on ice. Lysates were centrifuged at 18,000×*g* for 5 min at 4 °C. The resulting supernatants (~400 µL) were divided into 50 µL aliquots in new, precooled 1.5 mL tubes and stored at −80 °C. Total RNA concentration was quantified using the Qubit fluorometer. Each aliquot was used only once for downstream polysome profiling.

Linear 15 to 45% sucrose gradients were prepared using 10% and 50% (w/v) sucrose solutions in 50 mM Tris-HCl (pH 7.5) and supplemented with 100 mM NH_4_Cl, 10 mM MgCl_2_, and 6 mM β-mercaptoethanol. Solutions were sterile filtered and gradients generated using the Gradient Master (BioComp Instruments) following the manufacturer’s preprogrammed settings (SW60Ti rotor, short sucrose program, w/v 1st). Prepared gradients were stored at 4 °C overnight prior to use.

For polysome analysis, 50 µg of cell lysate was carefully layered onto each gradient without disturbing the interface. Samples were centrifuged at 40,000 rpm for 2 h and 30 min at 4 °C in an Optima L-100 K ultracentrifuge (Beckman Coulter) equipped with an SW60Ti rotor. Immediately following centrifugation, the sucrose gradients were carefully placed on ice to prevent disturbance, and continuous polysome absorbance profiles at 254 nm were recorded using the BioComp gradient fractionation system complemented with a TRIAX flow cell. 14 discrete fractions were sequentially collected from the top to the bottom of each gradient using the automated fraction collector, following the manufacturer’s instructions and stored at −80 °C until further analysis.

### Puromycin Assay.

To assess active translation, both wt *S. enterica* and *Y. pseudotuberculosis* strains were cultured as described above for overnight and subculture preparation. Upon reaching exponential phase (OD_600_ of 0.4), each culture was distributed into four separate flasks representing the following treatment conditions: 1) chloramphenicol (50 µg/mL), 2) unexposed control, 3) osmotic stress (0.5 M NaCl), or 4) low iron stress (250 µM 2,2′-Bipyridyl; Sigma-Aldrich). Cultures grew with their respective conditions for 10 min. Subsequently, to abruptly freeze and capture the translational landscape, both 10 µg/mL puromycin (P8833, Sigma-Aldrich) and 300 µg/mL chloramphenicol were simultaneously added to the culture, followed by an additional 10-min incubation period prior to cell lysis. The experiment was performed in four independent biological replicates for *Y. pseudotuberculosis* and three for *S. enterica.*

To extract proteins, 20 mL of bacterial culture was collected from each treatment group, band cells were immediately harvested by centrifugation at 4,000×*g* for 10 min at 4 °C. The resulting cell pellets of *Y. pseudotuberculosis* were washed twice with phosphate-buffered saline and resuspended in 1 ml of lysis buffer [100 mM Tris-HCl, 10 mM EDTA, 1× cOmplete™ protease inhibitor cocktail (Roche)]. The cell suspensions were lysed on ice using sonication for nine 20 s cycles, each cycle comprised 20 s sonication step separated by 30 s intervals with 10% amplitude. For *S. enterica*, lysozyme was additionally added into the lysis buffer at a final concentration of 100 µg/mL and incubated on ice for 30 min prior to sonication, as sonication alone was insufficient to achieve lysis. Following centrifugation at 13,000×*g* for 10 min at 4 °C, the supernatant was collected and stored at −20 °C. Protein concentrations were defined using QuBit Protein Assay Kit (Catalog No.: Q33211).

### Immunoblotting.

Protein concentrations were fixed across samples, and proteins were mixed in 1× sample buffer (2% SDS, 62.5 mM Tris-HCl, 20% glycerol, 0.05% bromophenol blue, 1% β-mercaptoethanol). Proteins were separated by sodium dodecyl sulfate (SDS)-PAGE on Mini-PROTEAN TGX gels (Bio-Rad) at 120 V for 1 h 10 min, loading 11 µg for *S. enterica* on 4 to 20% gels and 17 µg for *Y. pseudotuberculosis* on 4 to 15% gels. The SDS gel was transferred onto methanol activated Immobilon-P PVDF membrane (Millipore) by semidry electroblotting using Bio-Rad Trans-Blot SD Semi-Dry Transfer Cell (70 V for 1 h 10 min). Membranes were blocked with 5% milk in 1× Tris-buffered saline with Tween (TBST) and probed with Anti-Puromycin at 1:10,000 dilution (Sigma-Aldrich, MABE343, clone 12D10) primary antibody in 1× TBST, overnight at 4 °C. Following each antibody incubation, the membranes were washed three times for 10 min with 1× TBST. Membranes were incubated with secondary antibody: monoclonal Sheep Anti-Mouse IgG-Horseradish Peroxidase at 1:7,000 dilution (Cytiva, NA931V) in 1× TBST for 1 h at room temperature. The final blot was developed by chemiluminescence (Immobilon Western Chemiluminescent HRP Substrate, Millipore) for 5 min, and the bands were detected with Amersham Imager 680. Band intensities were quantified using ImageJ (version 1.54p).

#### Transcriptomic data retrieval and proteomic data generation.

The transcriptome data published for *S. enterica*, *Y. pseudotuberculosis*, and *S. aureus* were obtained from Gene Expression Omnibus (GEO) (73) with Accession No. GSE152295.

Proteomics data were obtained by exposing the three bacterial species to the same stress conditions as mentioned in Avican et al. (1), with unexposed control condition and excluding virulence inducing condition. After stress exposures, 3 mL of bacterial cultures were pelleted with 9,000 RPM for 2 min centrifugation and cell pellets were lysed in 200 µL 2% SDS. Thereafter, 5 μg of each sample were reduced with 20 mM TCEP. Samples were digested with a modified sp3 protocol (74), as previously described (43). Briefly, samples were added to a bead suspension (10 μg of beads (Sera-Mag Speed Beads, 4515-2105-050250, 6515-2105-050250) in 10 μL 15% formic acid and 30 μL ethanol) and incubated shaking for 15 min at room temperature. Beads were then washed four times with 70% ethanol. Proteins were digested overnight by adding 40 μL of 5 mM chloroacetamide, 1.25 mM TCEP, and 200 ng trypsin in 100 mM HEPES pH 8.5. Peptides were eluted from the beads and were dried under vacuum. Peptides were then labeled with TMT11plex (Thermo Fisher Scientific), pooled and desalted with solid-phase extraction using a Waters OASIS HLB μElution Plate (30 μm). Samples were fractionated into 48 fractions on a reversed-phase C18 system running under high pH conditions, with every sixth fraction being pooled together. Samples were analyzed by LC–MS/MS using a data-dependent acquisition strategy on a Thermo Fisher Scientific Vanquish Neo LC coupled with an Thermo Fisher Scientific Orbitrap Exploris 480. Raw files were processed with MSFragger using standard settings for TMT (75) against FASTA databases of each organism obtained from NCBI; for *S. enterica* Typhimurium SL1344 Accession Nos. NC_016810, NC_017718, NC_017719, and NC_017720; for *Y. pseudotuberculosis* Accession No. NC_010465; for *S. aureus* (MSSA476) Accession No. NC_002952. The mass spectrometry proteomics data have been deposited to the ProteomeXchange Consortium via the PRIDE partner repository (76) with the dataset identifier PXD055243.

### Data Processing and Normalization.

#### Processing of transcriptomic data.

Transcriptomic data were imported from PATHOgenex, including statistical values such as LogFC, *P*-value, and adjusted *P*-value. Moreover, the differential expression analysis was also already conducted, and thus no further preprocessing was done here.

#### Processing of proteomic data.

Raw protein files containing intensity data from TMT runs were imported into R (77) (version 4.4.2). Data were preprocessed to retain only protein locus tag, intensity values, and razor peptide count, defined as peptides shared across multiple proteins. Proteins were then filtered to retain only those quantified by at least two unique peptides and exhibiting nonzero intensity values; entries with zero intensity were considered missing data and excluded. This processing yielded one matrix per species per run, in which each row contained a protein identifier, its total intensity, and its corresponding razor peptide count.

To estimate protein abundance, each protein was mapped to its corresponding treatment condition. The relative reporter ion abundance of a given protein for its treatment was divided by the total relative reporter ion abundance as a first estimate of protein abundance. Given the large variation in protein length observed across the dataset—ranging from 10 to 3,000 amino acids in *S. enterica* and *Y. pseudotuberculosis*, and from 10 to approximately 2,000 amino acids in *S. aureus* (*SI Appendix*, Fig. S17), we applied a correction to account for size-dependent differences in peptide yield. Since longer proteins produce more tryptic peptides upon digestion, and consequently more ions, the initial estimate was divided by the number of expected tryptic peptides to obtain iBAQ (15). This ensures that iBAQ reflects protein abundance independently of protein size. Matrices from the same species were subsequently merged across runs, and proteins absent across all replicates were excluded, yielding three species-specific matrices each containing iBAQ values for every detected protein across the three biological replicates and their associated treatment conditions.

To identify the most appropriate normalization strategy, eight normalization methods were evaluated using the NormalyzerDE (45) package (version 1.23.2): VSN, Log_2_-transformation, Quantile, Global Intensity, Mean, Median, Linear Regression, and Cyclic Loess. Normalized outputs were visualized using principal component analysis (PCA) plots generated with ggplot2 (78) (version 4.0.2) to assess replicate clustering within each stress condition. VSN consistently produced the tightest replicate clustering across condition and was therefore applied to the three species-specific matrices to generate VSN-normalized iBAQ values for downstream analysis.

Differential abundance analysis was performed on the normalized proteome data using the limma (46) package (version 3.58.1) with default parameters, applying empirical Bayes moderation to stabilize variance estimates across proteins. Differentially abundant proteins were identified for each species to characterize the proteomic response to every stress condition, and highlight proteins potentially involved in key regulatory mechanisms.

### Data Analysis.

#### Correlation analysis.

To assess the correlation between mRNA and protein levels, we used the log_2_-transformed mean TPM values for mRNAs and the log_2_-transformed mean iBAQ values for proteins from three replicates of each condition. The Pearson correlation coefficient was calculated for each condition to determine the strength of the correlation. Statistical significance *P*-value < 0.05 was evaluated using Student’s *t* test.

#### Identification of gene clusters with discordant mRNA and protein level regulation.

To identify genes with discordant mRNA and protein level regulation under 10 stress conditions, the Z-score values of mRNA from control data were combined with the Z-score values of protein, calculated using limma differential expression analysis to create a single data table and visualize using scatter plots separately for each species. Thereafter, the combined transcriptome and proteome data were filtered with *P*-value < 0.05 to select genes whose mRNA or protein level changes upon exposure to stress conditions.

Genes were then grouped in 6 different clusters based on the expression patterns as described in *SI Appendix*, Table S1. Cluster 1: mRNA downregulated in at least one stress condition but protein level is not changed; Cluster 2: mRNA downregulated in at least one condition but protein is upregulated; Cluster 3: mRNA level is not changed in any condition but protein is downregulated; Cluster 4: mRNA level is not changed in any condition but protein is upregulated in at least one condition; Cluster 5: mRNA is upregulated in at least one condition but protein is downregulated; and Cluster 6: mRNA is upregulated in at least one condition but protein level is not changed.

#### Integrative omics analysis with MOBILE.

MOBILE pipeline (35) is used for three species with 11 samples including both transcriptomic and proteomic datasets. The iBAQ datasets were log_2_-transformed and normalized. First, they were filtered to only retain the most highly variant features (top 10% for transcripts and 20% for proteins). Next, the MOBILE pipeline (MATLAB version R2023a) is run, using the glmnet package (79) and Y=β.X+δ formalism, where *Y* matrix is the proteomics dataset and *X* is the transcriptomics. The objective is to obtain the coefficient matrix *β*. *δ* is the y-intercept values, which are almost zero by design. The regression process is repeated 10,000 times with randomly selected random number generation seeds to yield an ensemble of Lasso matrices. By identifying coefficients that appeared in at least half of the models, a robust Lasso coefficient matrix is selected. This matrix represents the stable associations between analytes across omics layers.

To find stress-specific interactions, MOBILE employs a “Leave-One-Group-Out” (LOGO) strategy where each sample exposed to the stress is excluded from the input one-at-a-time. Here, both the osmotic stress and control condition samples were subtracted, and corresponding final matrices were named as OSS-IAN and CTRL-IAN matrices. IANs are the Integrated Association Networks that are coalesced gene-level networks, where nodes represent genes of the matrix analytes and edges stand for the nonzero coefficients.

### Functional Annotation.

#### Enrichment analysis.

GO enrichment analyses were conducted using the clusterProfiler (80) package v4.10.1 in R. Most significant enriched terms were identified using a cut-off criterion of *P*-value < 0.05. Plots showing the most significant enriched Biological Process (BP) GO terms were generated using the ggplot2 package.

#### ORA.

The subsequent step involves comparing OSS-IAN and FULL-IAN (all condition matrix), to obtain OSS-dependent interactions. Similarly, comparing CTRL-IAN to FULL-IAN yields CTRL-dependent interactions. Next, pathway enrichment analysis (clusterProfiler version 4.14.6) was adopted for the genes list obtained by comparing OSS-dependent list to CTRL-dependent gene list. GO gene sets curated for three species were used in gmt format with a minimum gene set size of 15 and a maximum of 500. Significantly enriched gene sets were filtered by q-value < 0.2 and *P*-value < 0.05.

## Supplementary Material

Appendix 01 (PDF)

Dataset S01 (XLSX)

Dataset S02 (XLSX)

Dataset S03 (XLSX)

## Data Availability

All data necessary to reproduce the findings of this study are publicly available without restriction. The mass spectrometry proteomics data have been deposited to the ProteomeXchange Consortium via the PRIDE partner repository (76) with the dataset identifier PXD055243. The transcriptome data published for *S. enterica*, *Y. pseudotuberculosis*, and *S. aureus* were obtained from PATHOgenex RNA Atlas (1) with Gene Expression Omnibus (GEO) (73) Accession No. GSE152295. The complete dataset is available on Zenodo (81) (https://zenodo.org/records/19488690). A repository containing a Rnotebook with all the code to reproduce the figures and analyses presented in this manuscript is available on GitHub at https://github.com/avicanlab/RNAProtCorr. All packages and versions are listed in the Renv file available in the GitHub repository.
