# Supplementary material for: Bacterial stress responses lower mRNA–protein level correlations
Source: Proc Natl Acad Sci U S A. 2026 Sep 16;123(38):e2613102123. doi: 10.1073/pnas.2613102123 (PMC13598236; doi:10.1073/pnas.2613102123)
Supplement: Supplementary file 1 [file pnas.2613102123.sapp.pdf]

Updated August 2025

PNAS

# Supporting Information for

## Bacterial Stress Responses Lower mRNA-Protein Level Correlations.

Sena G. Süer[1,2,3,4†], Jérôme Arnoux[1,2,3,4†], Yi Y. Lim[1,2], Ganeshwari Dhurve[1,5], Rabia Şen[4,6], Cemal Erdem[4,6], André Mateus[3,4,5,7] and Kemal Avican[1,2,3,4,8*]

1. Department of Molecular Biology, Umeå University, Umeå, 90187, Sweden.
2. Integrated Science Lab (IceLab), Umeå University, Umeå, 90187, Sweden.
3. Umeå Centre for Microbial research (UCMR), Umeå University, Umeå, 90187, Sweden.
4. Science for Life Laboratory (SciLifeLab), Umeå University, Umeå, 90187, Sweden.
5. Laboratory for Molecular Infection Medicine Sweden (MIMS), Umeå University, Umeå, 90187, Sweden.
6. Department of Medical Biosciences, Umeå University, Umeå, 90187, Sweden.
7. Department of Chemistry, Umeå University, Umeå, 90187, Sweden.
8. Lead contact

†These authors share first authorship.

*Correspondence: Kemal Avican

**Email:** kemal.avican@umu.se

**Author Contributions:** K.A. designed research; S.G.S., JA, Y.Y.L., G.D., R.Ş., C.E., A.M and K.A performed research; S.G.S., and G.D. contributed new reagents/analytic tools; S.G.S., J.A., Y.Y.L., R.Ş., C.E., A.M and K.A. analyzed data; and S.G.S., J.A., and K.A. wrote the paper with input from G.D., R.Ş., C.E., A.M

**Competing Interest Statement:** The authors declare no competing interests.

**Classification:** BIOLOGICAL SCIENCES, Microbiology; Systems Biology

**Keywords:** mRNA-protein correlation, infection, stress response, pathogen.

**This PDF file includes:**

Supporting text
Figures S1 to S17
Table S1

**Supporting Information Text**

All data necessary to reproduce the findings of this study are publicly available without restriction. The complete dataset is available from Zenodo (https://zenodo.org/records/19488690). Transcriptomic data were obtained from PATHOgenex RNA Atlas; Protein sequences and Mass spectrometry results are provided in the Zenodo repository.

All code and commands necessary to reproduce the analyses are publicly available. A repository containing an R notebook with all code to reproduce the figures and analyses presented in this manuscript is available on GitHub at https://github.com/avicanlab/RNAProtCorr. All packages and versions are listed in the renv file available in the GitHub repository.

**Figures**

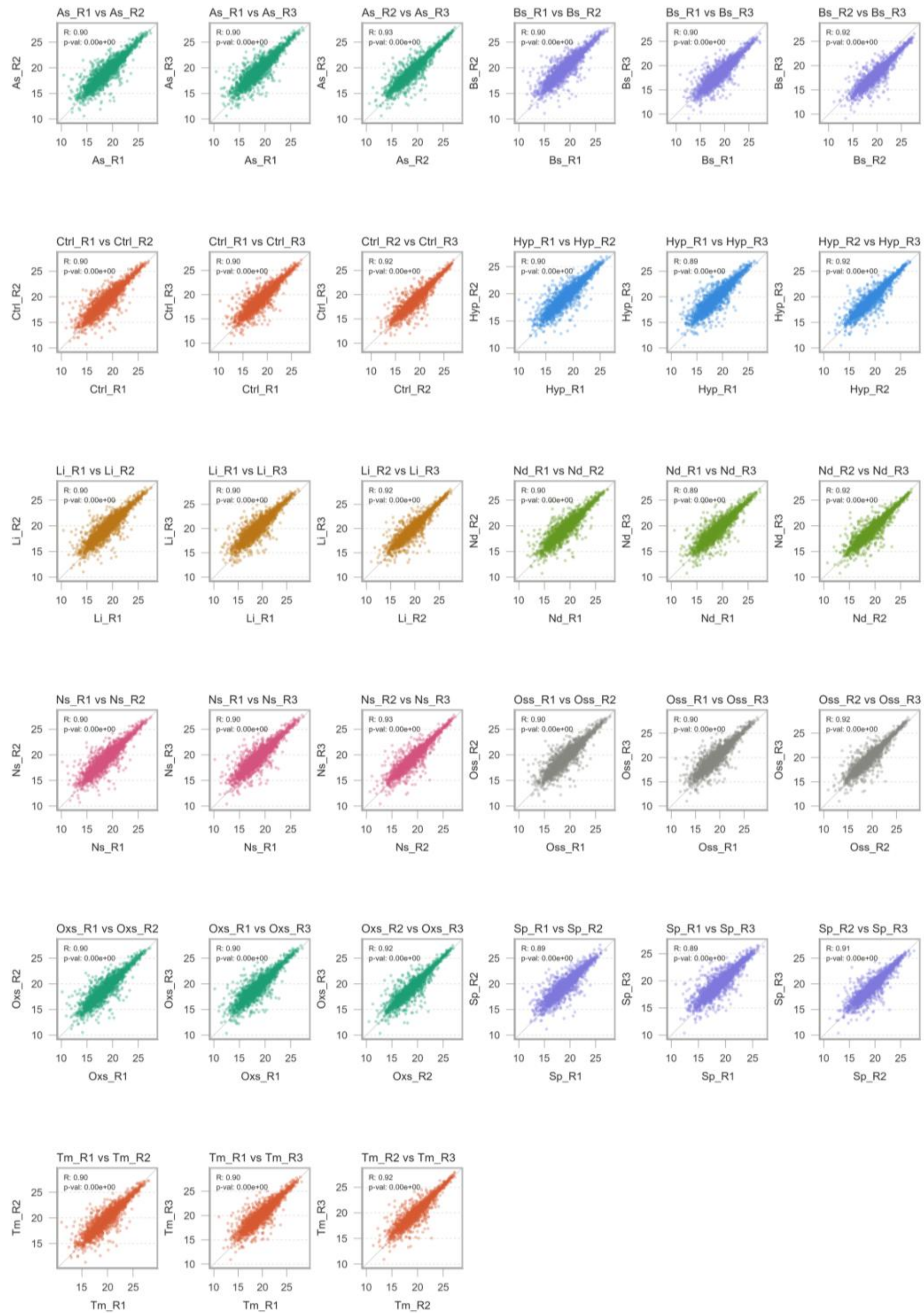


**Fig. S1. Replicates each condition show similar global expression patterns in *S. enterica* Typhimurium.** Pairwise comparison of $log_2$-transformed protein expression values (iBAQ) from each of the three replicates per condition were performed using proteomic analysis. The Pearson correlation coefficient (R) was used to measure similarity, and the Student's T-test was used to calculate the *p*-value.

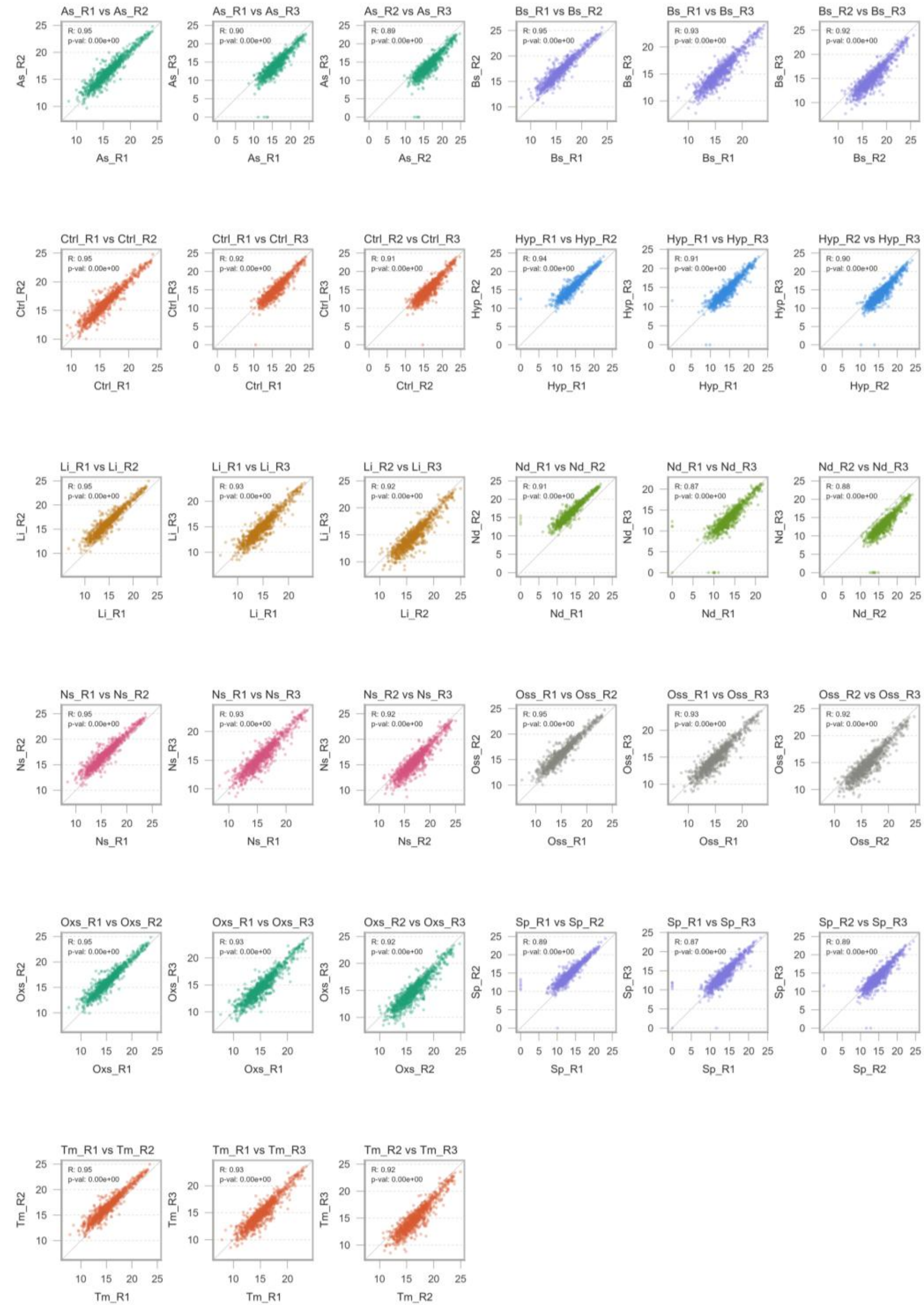


**Fig. S2. Replicates each condition show similar global expression patterns in *S. aureus*.** Pairwise comparison of $log_2$-transformed protein expression values (iBAQ) from each of the three replicates per condition were performed using proteomic analysis. The Pearson correlation coefficient (R) was used to measure similarity, and the Student's T-test was used to calculate the *p*-value.

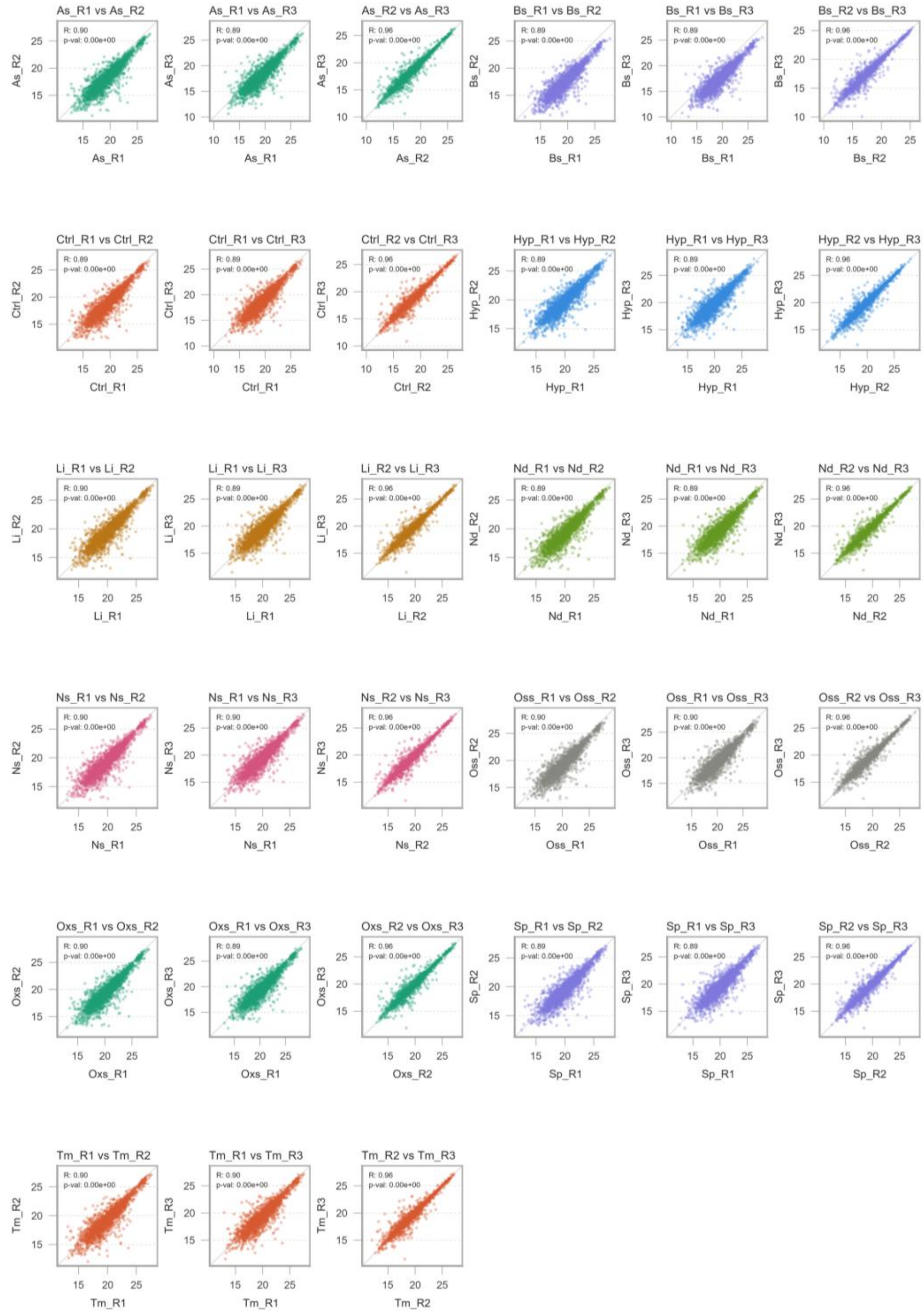


**Fig. S3. Replicates each condition show similar global expression patterns in *Y. pseudotuberculosis*.** Pairwise comparison of $log_2$-transformed protein expression values (iBAQ) from each of the three replicates per condition were performed using proteomic analysis. The Pearson correlation coefficient (R) was used to measure similarity, and the Student's T-test was used to calculate the *p*-value.

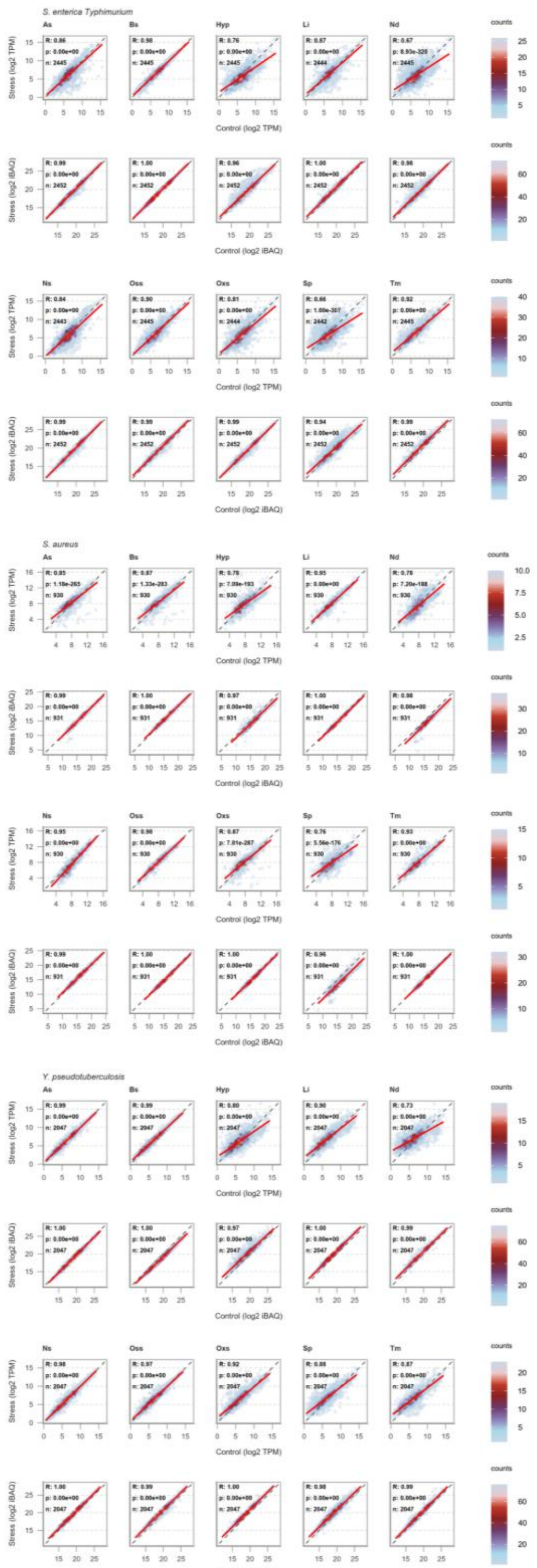


**Fig. S4. Control versus stress mRNA and protein levels for all genes.** mRNA and protein expression levels were taken as $log_2$-transformed TPM and iBAQ values, respectively. Top row represents the RNA expression level and bottom line the protein level. The expression values of genes that are detected in all conditions as mRNA and protein were used in this analysis. Pearson correlation coefficient (R) shows positive correlation under all conditions in the three species. *p*-values were calculated using Student's t-test.

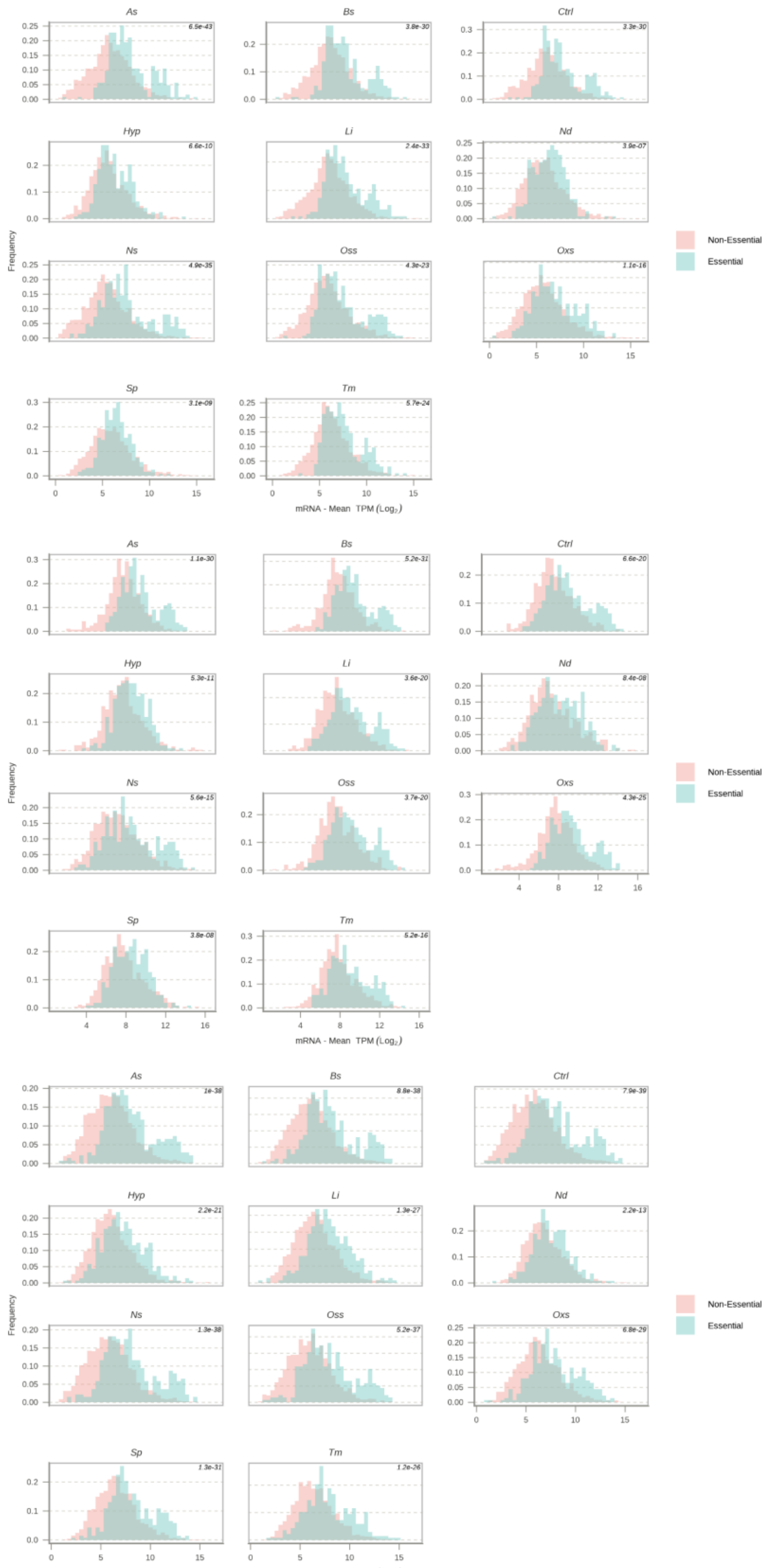


**Fig. S5. mRNA expression of essential genes are higher than non-essential genes in all tested conditions for the three species. A)** Frequency of gene expression levels in $log_2$-transformed TPM values in *S. enterica* Typhimurium **B)** *S. aureus* **C)** *Y. pseudotuberculosis*.

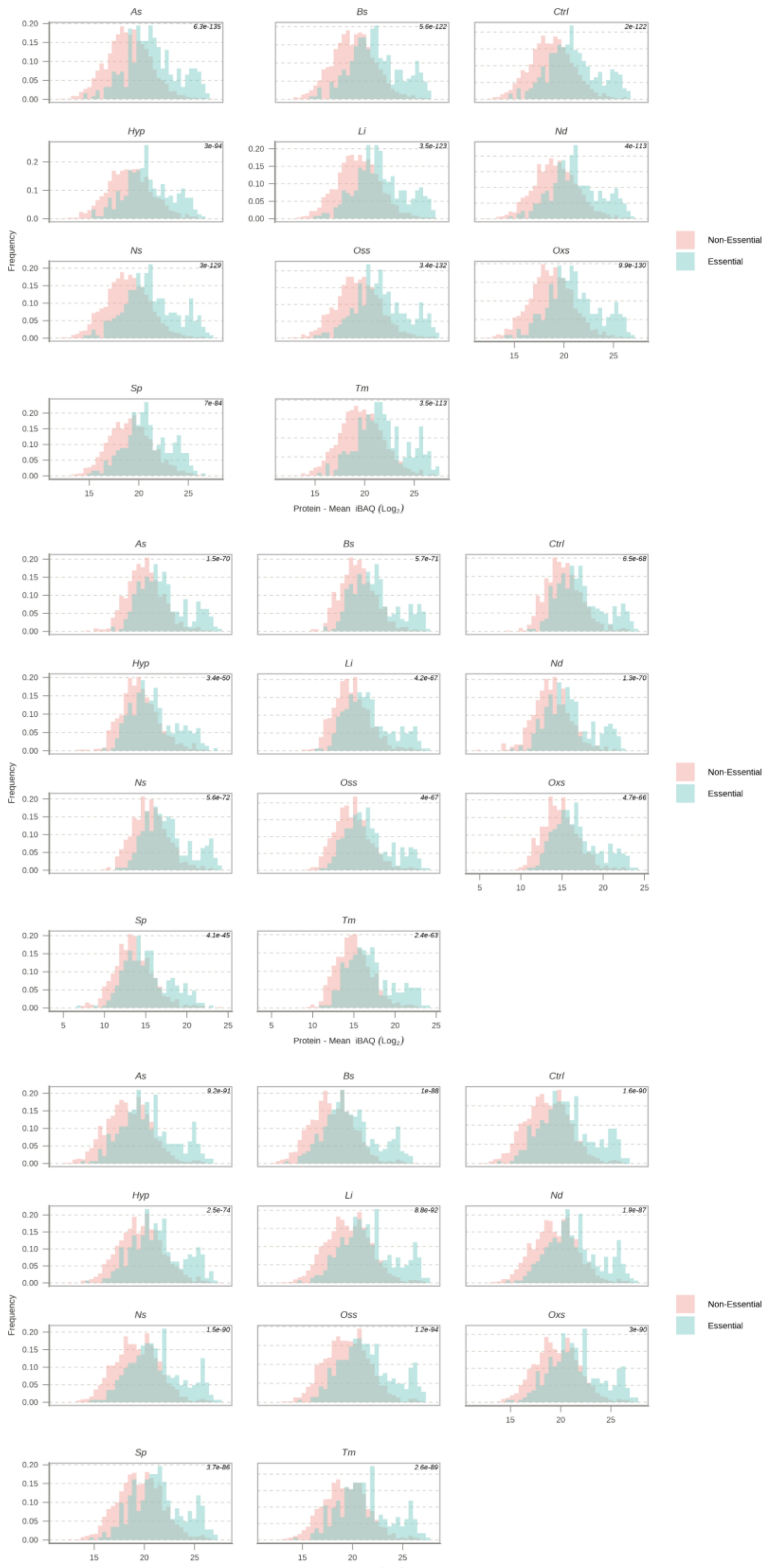


**Fig. S6. Protein expression of essential genes are higher than non-essential genes in all tested conditions for the three species. A)** Frequency of gene expression levels in $log_2$-transformed iBAQ values in *S. enterica* Typhimurium **B)** *S. aureus* **C)** *Y. pseudotuberculosis*.

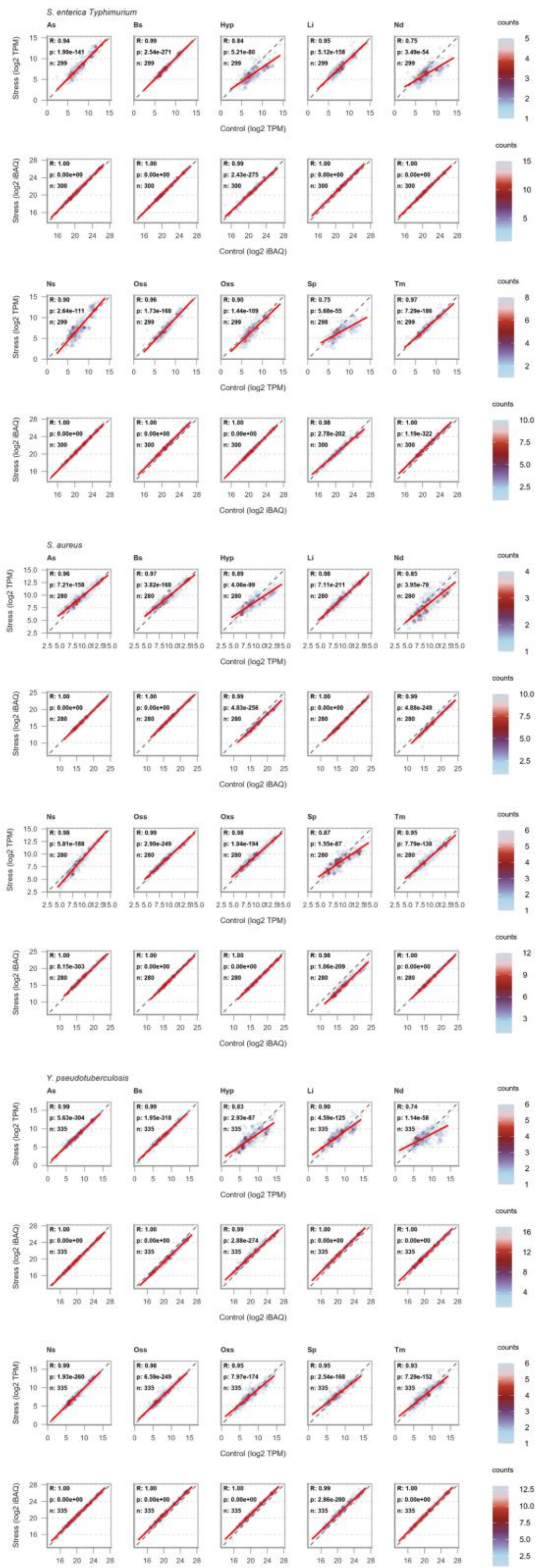


**Fig. S7. Control versus stress mRNA and protein levels for all genes.** mRNA and protein expression levels were taken as $\log_2$-transformed TPM and iBAQ values, respectively. Top row represents the RNA expression level and bottom line the protein level. The expression values of genes that are detected in all conditions as mRNA and protein were used in this analysis. Pearson correlation coefficient (R) shows positive correlation under all conditions in the three species. *p*-values were calculated using Student's t-test.

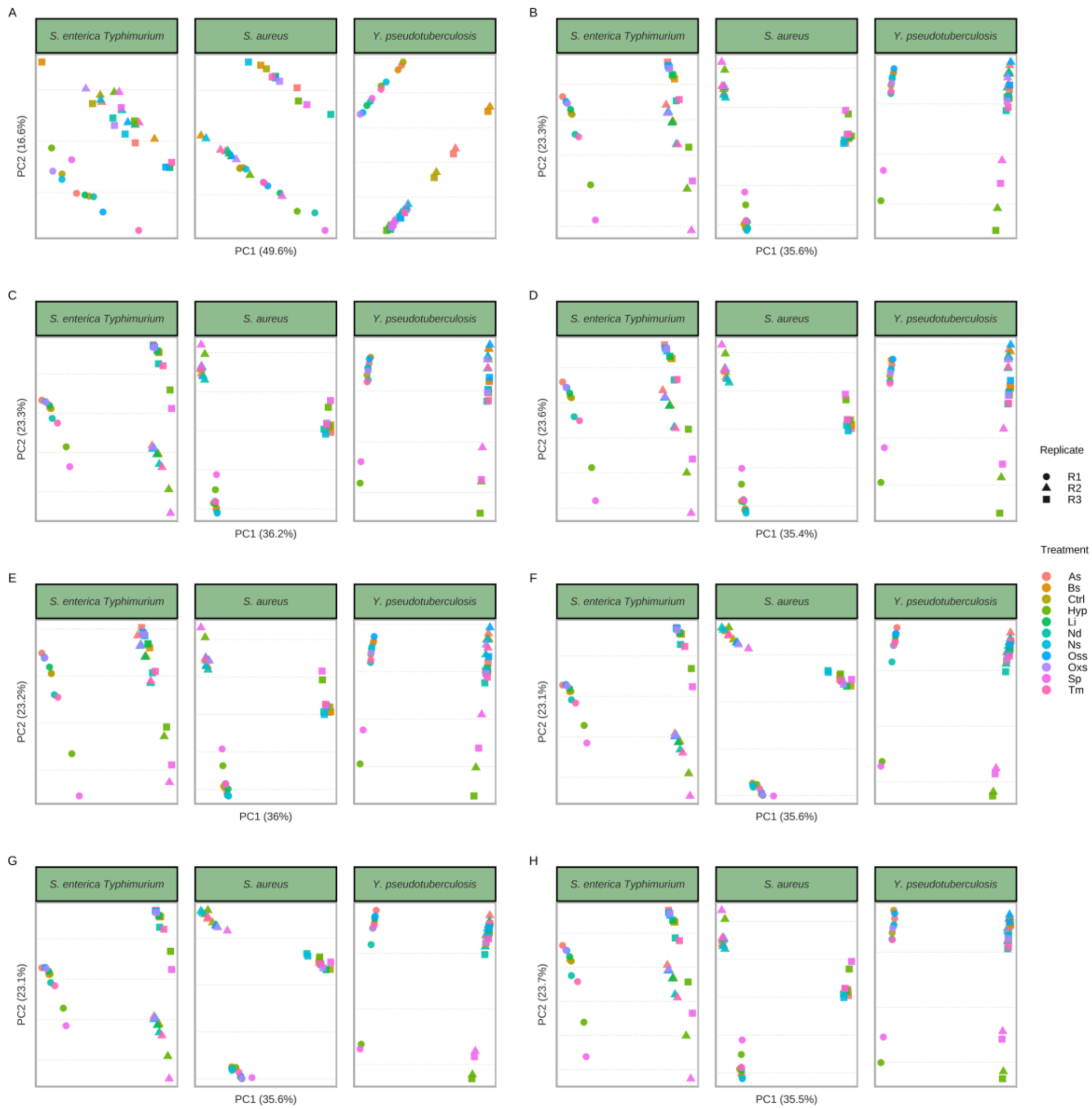


**Fig. S8. Variance Stabilizing Normalization (VSN) method performs better in clustering replicates from the same condition and separation between different conditions for proteome data from *S. enterica* Typhimurium, *S. aureus*, *Y. pseudotuberculosis*.** Principle Component Analysis (PCA) results of proteome data using different normalization methods are shown in panels for: **A)** $Log_2$, **B)** VSN, **C)** CycLoess, **D)** Quantile, **E)** Median, **F)** Mean, **G)** Global Intensity, **H)** Linear regression.

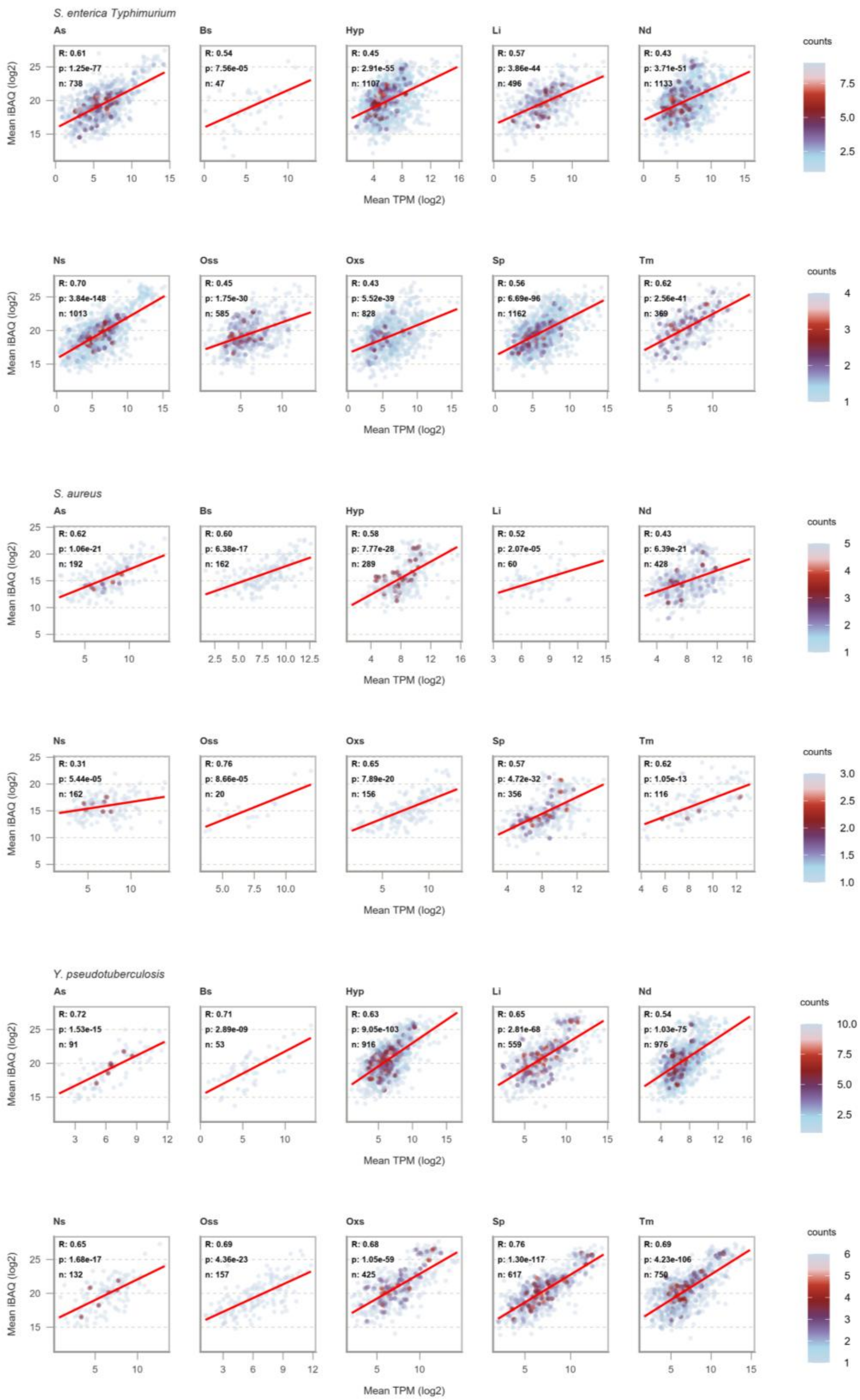


**Fig. S9. mRNA-protein levels of differentially expressed genes.** Correlations are measured under all stress conditions in *S. enterica* Typhimurium, *S. aureus* and *Y. pseudotuberculosis*. mRNA and protein expression levels were taken as $\log_2$ transformed TPM and iBAQ values, respectively. *p*-values were calculated using Student's t-test.

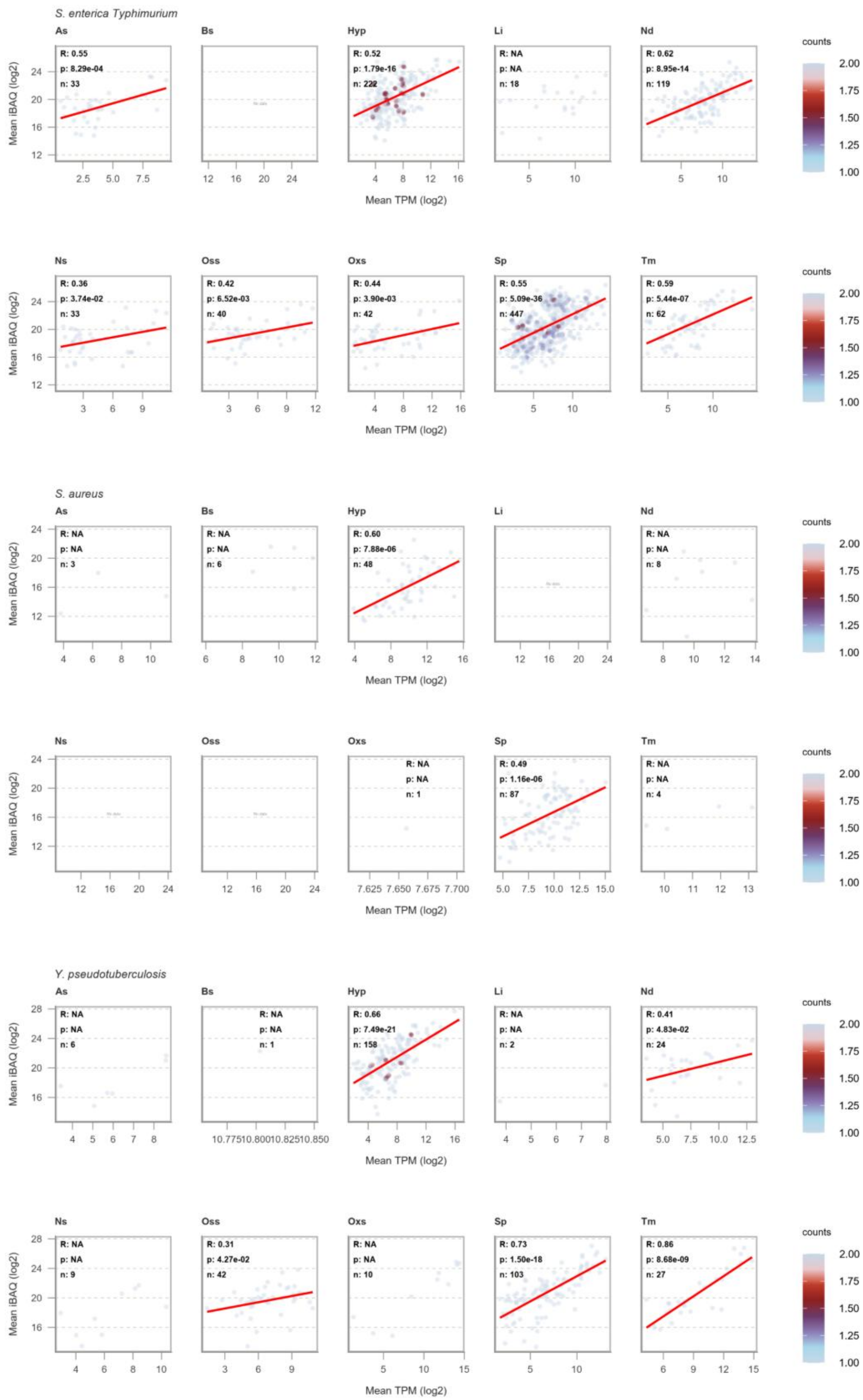


**Fig. S10. mRNA-protein levels of differentially expressed proteins.** Correlations are measured under all stress conditions in *S. enterica* Typhimurium, *S. aureus* and *Y. pseudotuberculosis*. mRNA and protein expression levels were taken as $log_2$ transformed TPM and iBAQ values, respectively. *p*-values were calculated using Student's t-test.

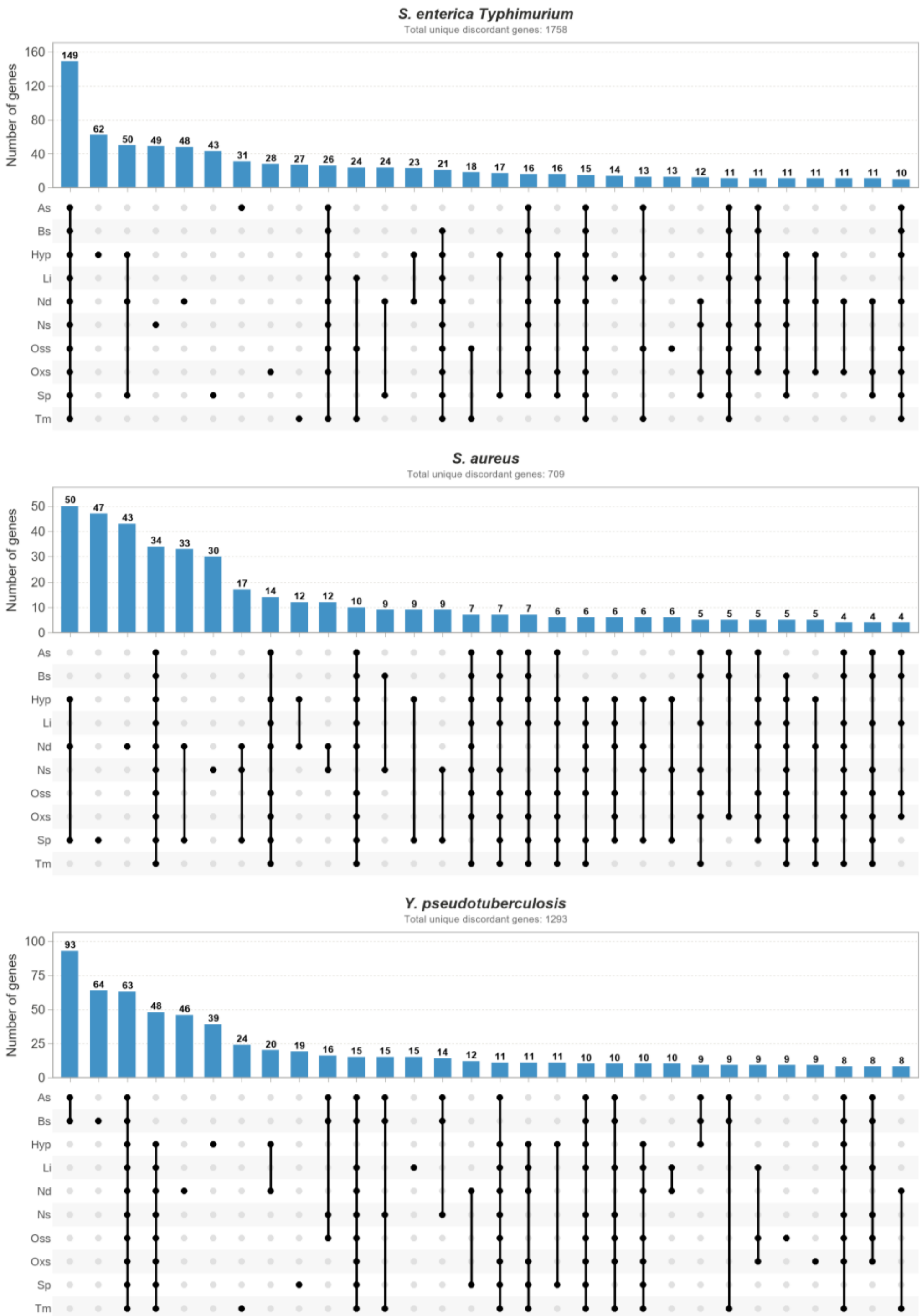


**Fig. S11. Recurrence of discordantly regulated genes across stress treatments.** Each bar represents the number of genes displaying discordant mRNA–protein regulation in a specific combination of stress treatments, ordered from most to least frequent (left to right). The combination matrix below each plot indicates which treatments (rows, ordered alphabetically) contribute to each intersection. Genes appearing in a single column are discordant exclusively under that condition, while genes in multi-treatment columns are recurrently discordant across conditions. The total number of unique discordant genes per species is indicated in the subtitle. Results are shown separately for *S. enterica* Typhimurium*, S. aureus, and Y. pseudotuberculosis*.

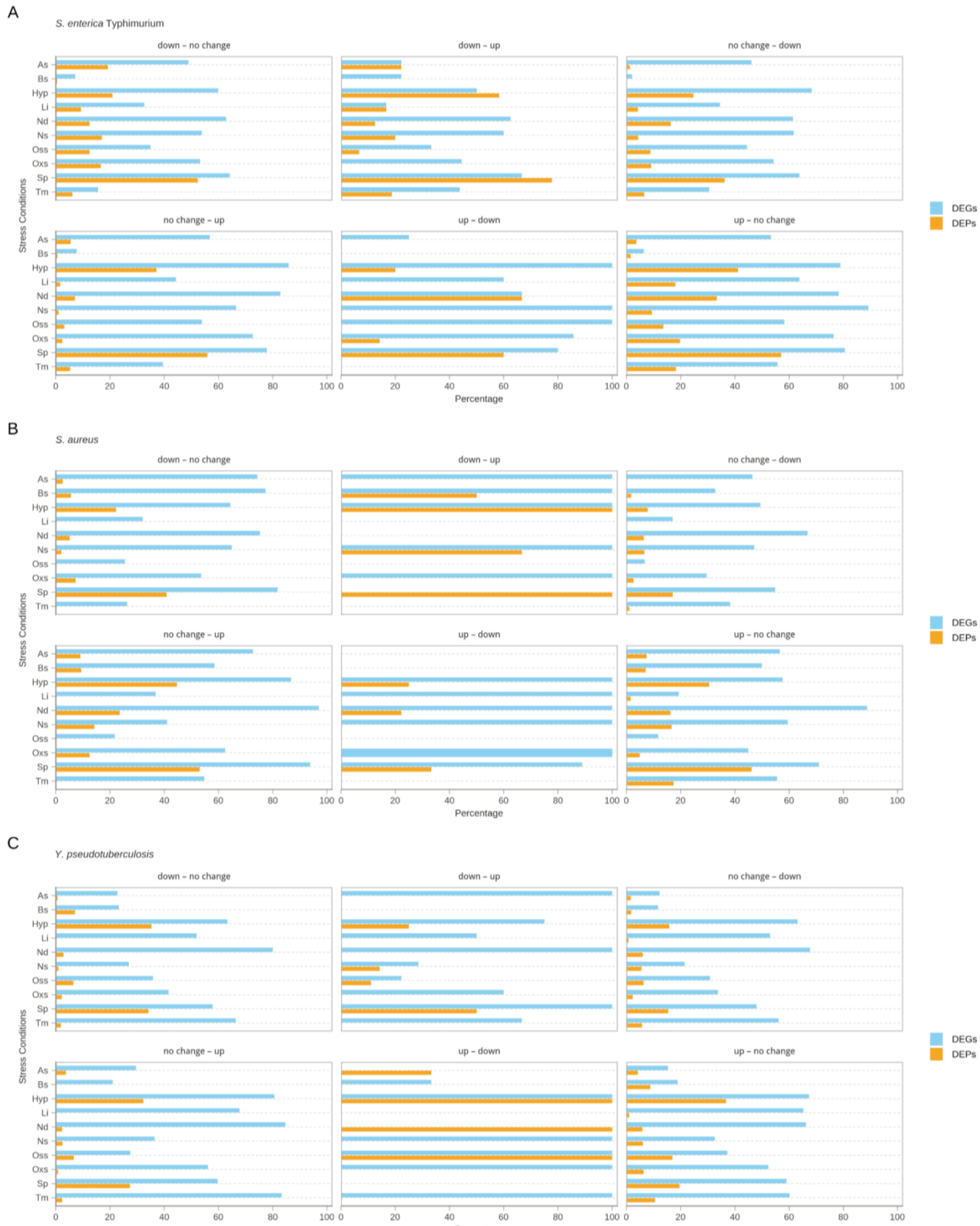


**Fig. S12. Genes with discordantly regulated mRNA and protein levels are found in all tested stress conditions for the three species.** Percentage of DEGs and DEPs, filtered with *p*-values<0.05, in each of the 6 discordantly regulated gene clusters for every tested stress conditions are shown as bars for **A)** *S. enterica* Typhimurium, **B)** *S. aureus*, **C)** *Y. pseudotuberculosis.*

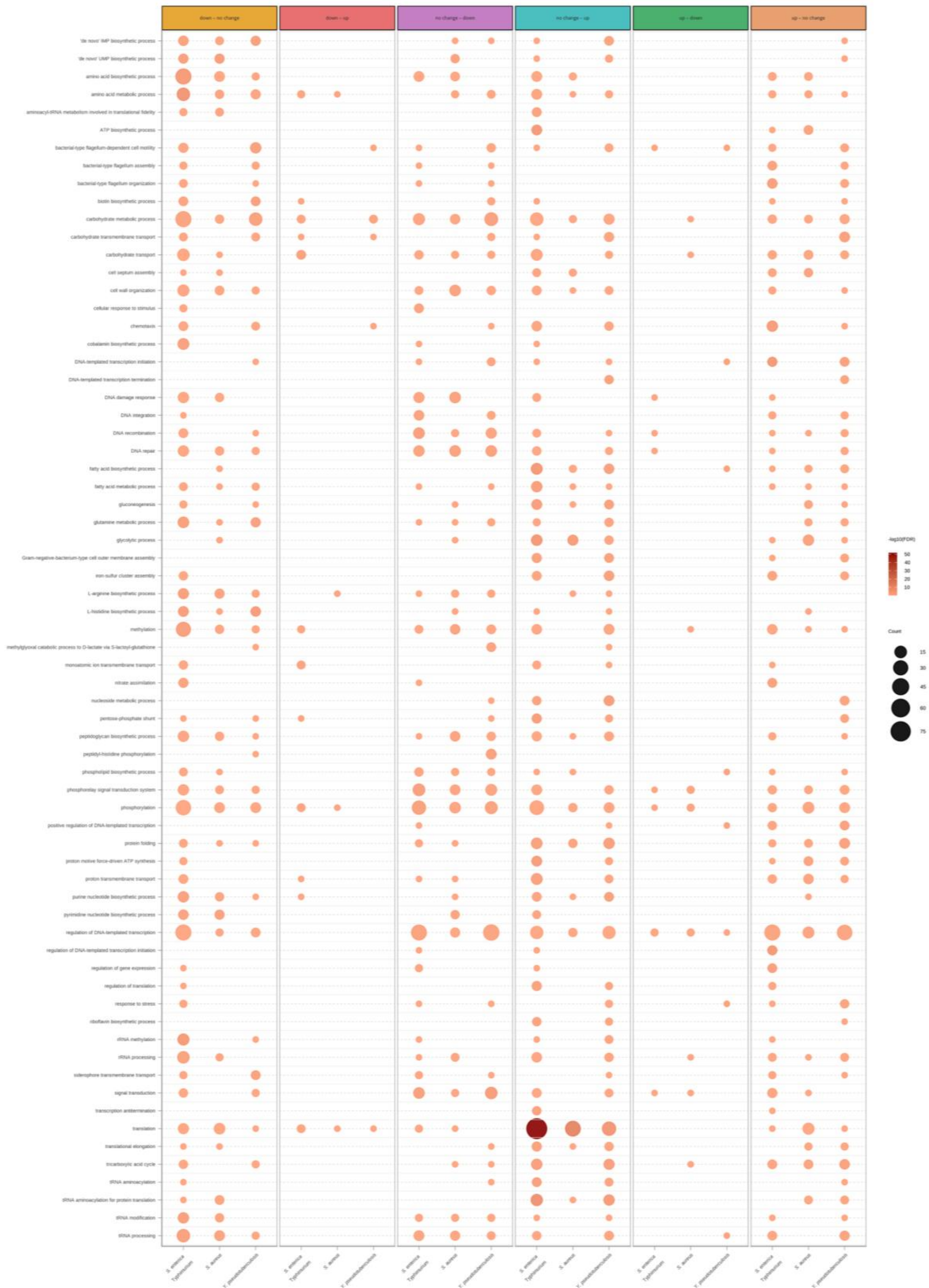


**Fig. S13. Stress response induces different patterns of discordant mRNA and protein level regulation highlighting key role for translation.** GO biological process enrichment analysis of 6 discordantly regulated gene clusters for the three species.

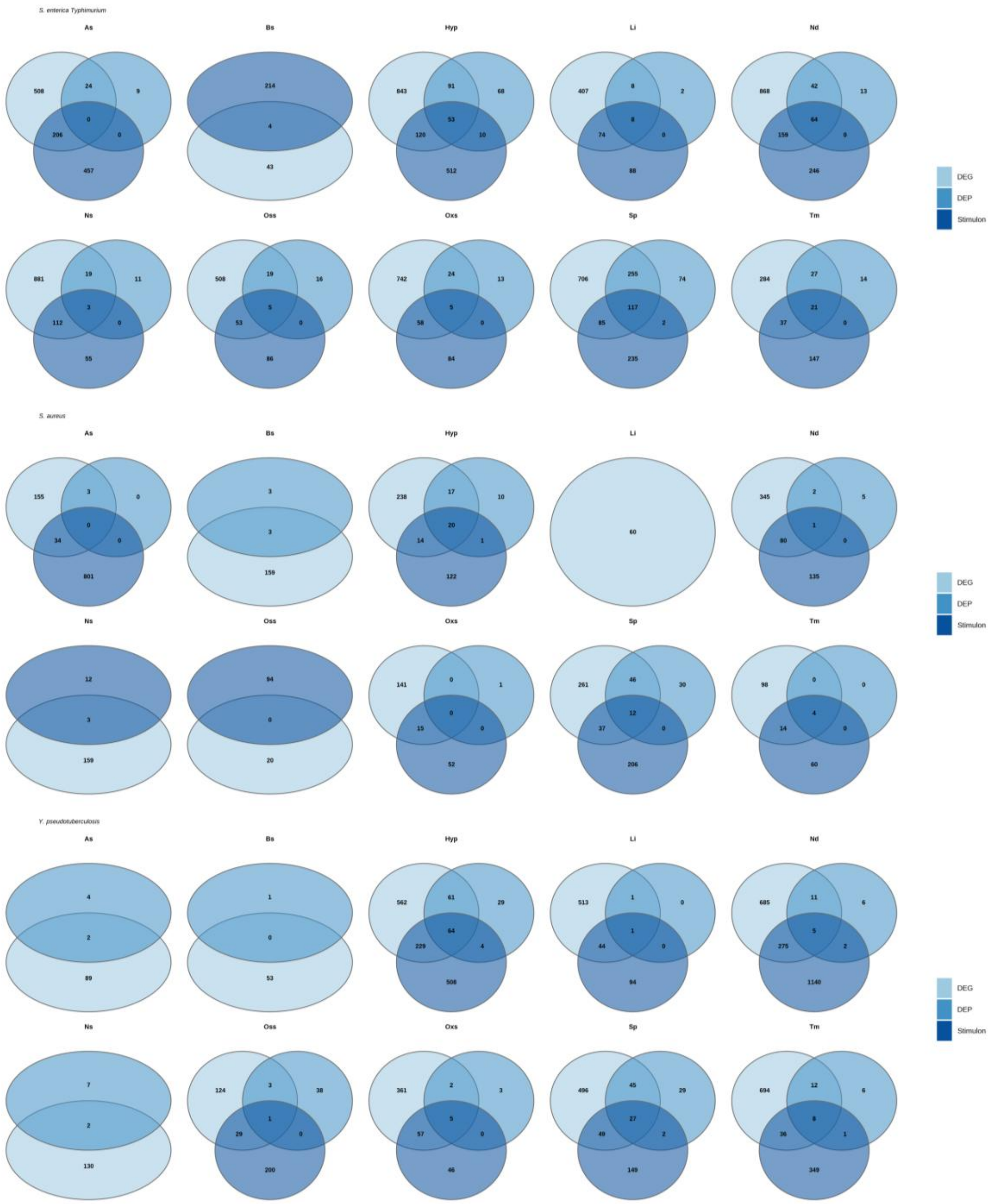


**Fig. S14. Venn diagrams showing the overlap between DEG, DEP and stimulon genes.** DEGs are colored in light blue, DEPs in medium blue, and stimulon in dark blue, for each stress treatment and species (*S. enterica* Typhimurium, *S. aureus*, and *Y. pseudotuberculosis*). Numbers indicate gene counts in each region. Panels with a single circle represent conditions where only one gene set was identified.

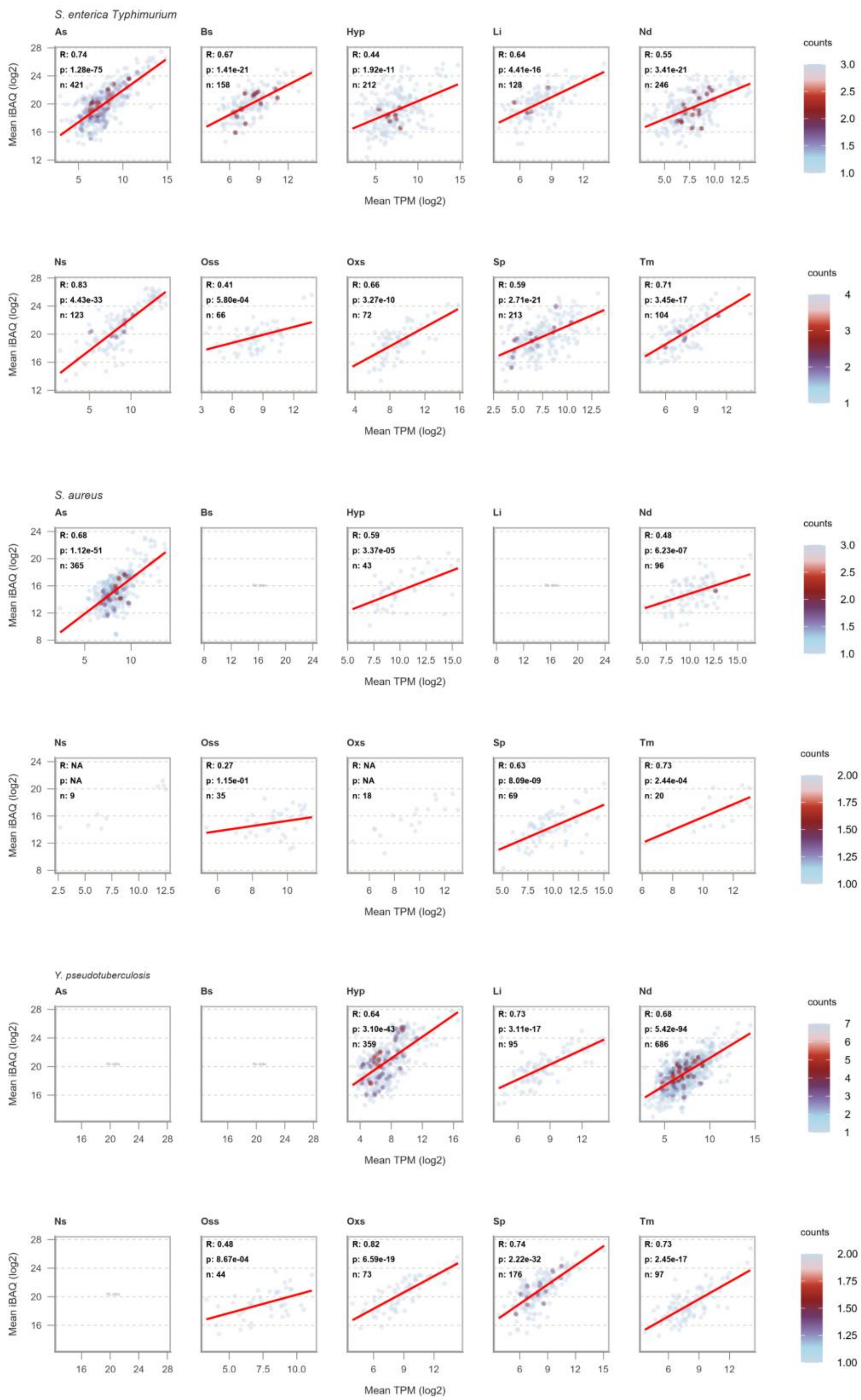


**Fig. S15. mRNA-protein levels of differentially expressed stimulon genes.** Correlations are measured under all stress conditions in *S. enterica* Typhimurium, *S. aureus* and *Y. pseudotuberculosis*. mRNA and protein expression levels were taken as $\log_2$ transformed TPM and iBAQ values, respectively. *p*-values were calculated using Student's t-test.

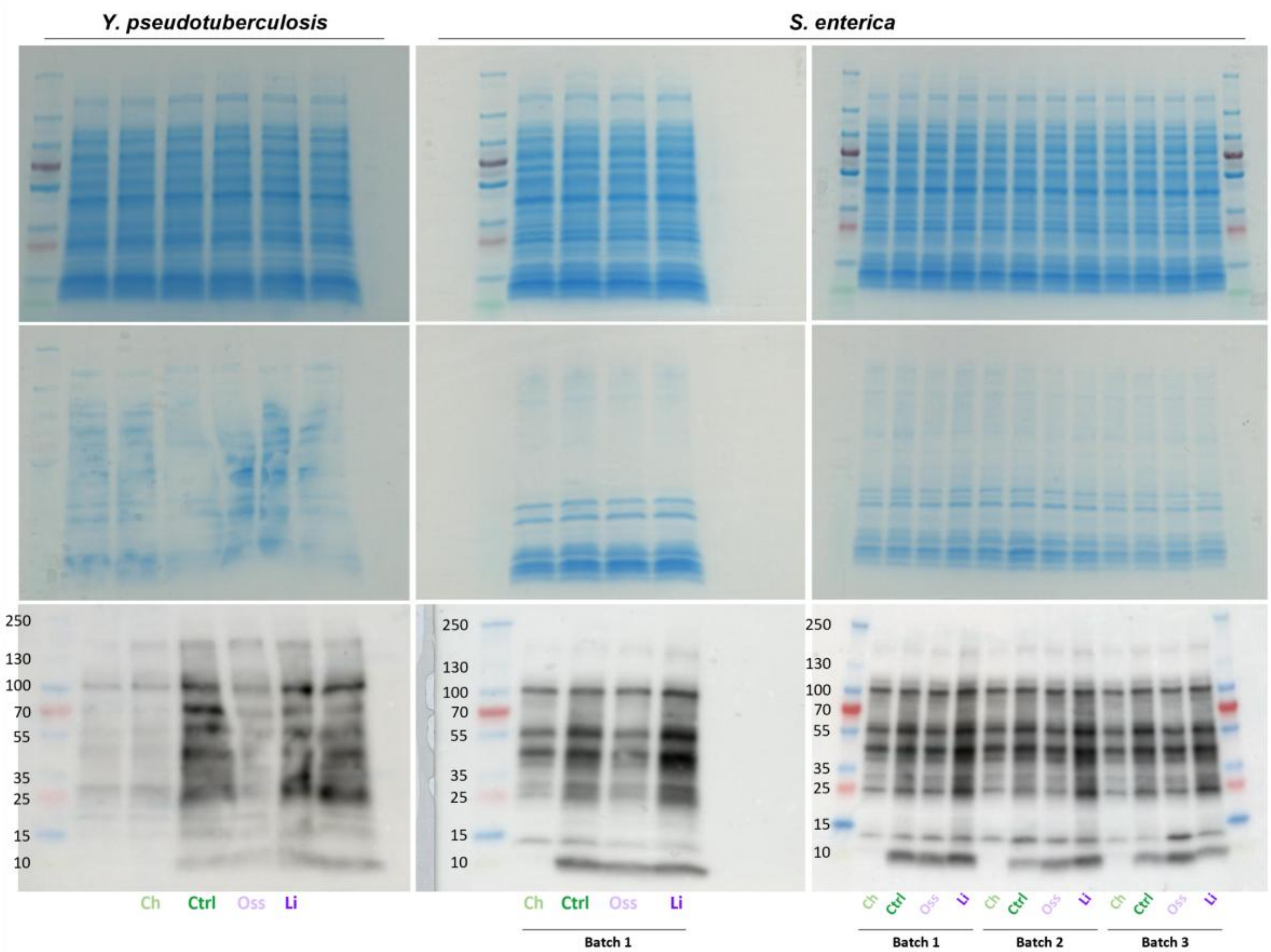


**Fig. S16. Translation efficiency is reduced under osmotic stress across biological replicates. A)** Western blot membranes showing active translation of nascent proteins detected using monoclonal anti-puromycin antibody in wt *Y. pseudotuberculosis* and *S. enterica* cells under negative control (Ch), positive control (Ctrl), osmotic stress (Oss), and low iron (Li) conditions. Three independent biological replicates are shown.

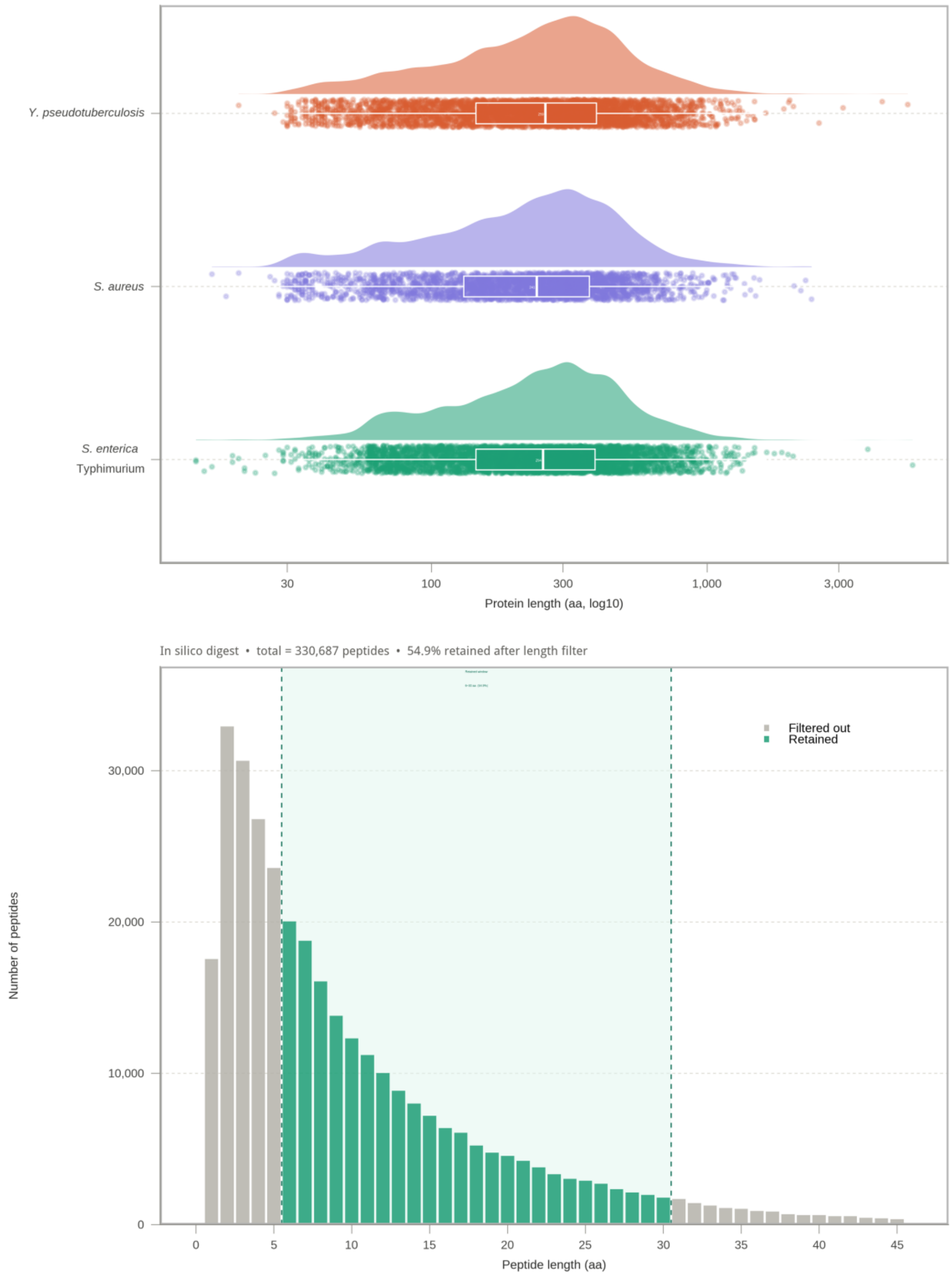


**Fig. S17. Protein and peptide length vary substantially within and across species.** A) Distribution of protein lengths across the three bacterial pathogens. Each point represents an individual protein, plotted on a log10-scaled x-axis. The shaded cloud reflects the density of proteins at each length, and the boxplot displays the median along with the first and third quartiles. B) Distribution of peptide lengths generated by trypsin digestion. Bars represent the density of peptides at each length. Peptides highlighted in green are those retained for iBAQ quantification, corresponding to tryptic peptides that fall within the expected detection range.

**Table**

**Table S1.** Classification of differentially expressed genes based on mRNA and protein regulation patterns.

| Cluster | mRNA regulation | Protein regulation | Description |
|---|---|---|---|
| 1 | ▼ Downregulated | ● Not changed | mRNA is downregulated in at least one stress condition, while protein level remains unchanged |
| 2 | ▼ Downregulated | ▲ Upregulated | mRNA is downregulated in at least one condition, while protein level is upregulated |
| 3 | ● Not changed | ▼ Downregulated | mRNA level remains unchanged across all conditions, while protein is downregulated |
| 4 | ● Not changed | ▲ Upregulated | mRNA level remains unchanged across all conditions, while protein is upregulated in at least one condition |
| 5 | ▲ Upregulated | ▼ Downregulated | mRNA is upregulated in at least one condition, while protein level is downregulated |
| 6 | ▲ Upregulated | ● Not changed | mRNA is upregulated in at least one condition, while protein level remains unchanged |
